# Computational exploration of copper catalyzed vinylogous aerobic oxidation of unsaturated compounds

**DOI:** 10.1038/s41598-020-80188-2

**Published:** 2021-01-14

**Authors:** Ting Wang, Yu Zhou, Yao Xu, Gui-Juan Cheng

**Affiliations:** 1 Warshel Institute for Computational Biology, Shenzhen Key Laboratory of Steroid Drug Development, School of Life and Health Sciences, The Chinese University of Hong Kong (Shenzhen), Shenzhen, 518172 China; 2 School of Life Sciences, University of Science and Technology of China, Hefei, 230027 Anhui China

**Keywords:** Chemistry, Physics

## Abstract

Selective oxidation is one of the most important and challenging transformations in both academic research and chemical industry. Recently, a highly selective and efficient way to synthesize biologically active *γ*-hydroxy-*α*,*β*-unsaturated molecules from Cu-catalyzed vinylogous aerobic oxidation of *α*,*β*- and *β*,*γ*-unsaturated compounds has been developed. However, the detailed reaction mechanism remains elusive. Herein, we report a density functional theory study on this Cu-catalyzed vinylogous aerobic oxidation of *γ*,*γ*-disubstituted *α*,*β*- and *β*,*γ*-unsaturated isomers. Our computational study unveils detailed mechanism for each elementary step, i.e. deprotonation, O_2_ activation, and reduction. Besides, the origin of regioselectivity, divergent reactivities of substrates as well as reducing agents, and the byproduct generation have also been investigated. Notably, the copper catalyst retains the + 2 oxidation state through the whole catalytic cycle and plays essential roles in multiple steps. These findings would provide hints on mechanistic studies and future development of transition metal-catalyzed aerobic oxidation reactions.

## Introduction

Selective oxidation has gained a preeminent position in both academic research and chemical industry^1–3^. One particular class of selective oxidation reactions achieved by a combined use of air as an oxidant and copper as a catalyst is highly desirable due to the natural abundance of air and copper^4–6^. Over the past few decades, great progress has been made in this field and many successful Cu-catalyzed aerobic reactions have been developed^7–23^.

Catalytic vinylogous reactions are among the most important reactions in organic synthesis due to their extensive application in the synthesis of complex natural products and bioactive molecules^24–27^. Despite significant advances in transition metal-catalyzed *α*- or *β*-functionalization of *α*,*β*- and *β*,*γ*-unsaturated compounds^28–32^, the vinylogous version leading to synthetically valuable *γ*-substituted *α*,*β*-unsaturated compounds^33–36^ has been rarely studied. For example, the vinylogous hydroxylation of *α*,*β*-unsaturated or *β*,*γ*-unsaturated compounds is a direct method to synthesize *γ*-hydroxy-*α*,*β*-unsaturated compounds^37–42^ which are valuable biological active pharmaceuticals and important intermediates in organic synthesis. However, the catalytic aerobic vinylogous hydroxylation is highly challenging due to the control of the reaction selectivity^43,44^, such as regioselectivity, chemoselectivity (hydroxylation vs*.* oxidative fragmentation, epoxidation, and other competitive oxidation reactions) and overoxidation problems.

**Scheme 1:**
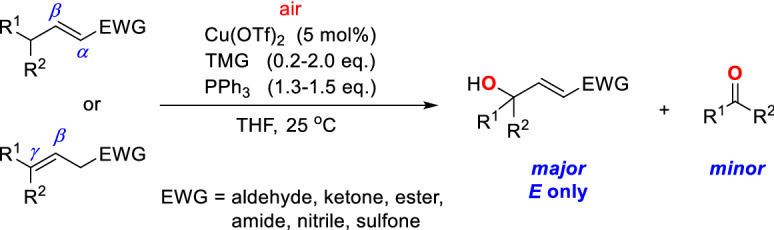
Copper-catalyzed vinylogous aerobic oxidation of *γ*,*γ*-disubstituted *α*,*β*- and *β*,*γ*-unsaturated compounds.

In 2018, Yin and Newhouse’s group successfully realized an efficient and operationally simple copper-catalyzed vinylogous oxidation reaction by using air as an oxidant, which leads to a broad array of *γ*-hydroxy-*α*,*β*-(*E*)-unsaturated compounds^45^. Reactions of both *γ*,*γ*-disubstituted *α*,*β*- and *β*,*γ*-unsaturated compounds produce *γ*-hydroxy-*α*,*β*-(*E*)-unsaturated compounds in high yield with perfect stereo- and regioselectivity (Scheme 1). The copper(II) triflate catalyst, the base (tetramethylguanidine, TMG), and the reducing agent (PPh_3_) were found essential for the reaction. Their method was successfully applied to the vinylogous oxidation of unsaturated esters, aldehydes, ketones, amides, nitriles, and sulfones, demonstrating great potential in the synthesis of natural products and bioactive molecules.

**Scheme 2:**
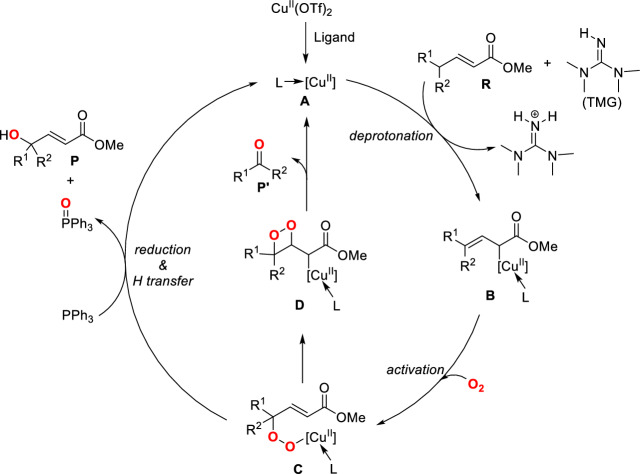
Proposed reaction mechanism for the copper-catalyzed vinylogous aerobic oxidation.

The preliminary mechanistic study indicated that radicals might not be involved in the reaction because the reaction efficiency was not affected by the addition of radical scavengers^46–48^. Based on the experimental observations, Yin et al*.* proposed a three-step pathway for the generation of the main *γ*-hydroxylated product (Scheme 2). This pathway consists of deprotonation (**A** + **R** + TMG → **B**), O_2_ activation (**B** + O_2_ → **C**) and PPh_3_ participated reduction step (**C** + PPh_3_ → **P** + P(O)Ph_3_). The ketone side-product **P′** was proposed to be formed through a four-membered endoperoxide intermediate **D**. Scheme 2 provides a general mechanism for this Cu-catalyzed aerobic vinylogous oxidation reaction, but the details of each elementary step are unknown. Besides, the origin of regioselectivity and the role of copper catalyst remain elusive. Furthermore, the inertness of *γ*,*γ*-dialkyl-substituted *α*,*β*-unsaturated compounds toward the aerobic oxidation reaction and the ineffectiveness of P(OEt)_3_ as reducing agent have not been fully understood. The understanding of the reaction mechanism is essential for further reaction development. Herein, we perform density functional theory (DFT) calculations^11^ on this Cu-catalyzed vinylogous aerobic oxidation reaction to elucidate the detailed reaction mechanism, to explore the role of copper catalyst and phosphine, and to understand the observed regioselectivity and side-product formation.

## Results and discussion

### The resting state of copper catalyst

**Scheme 3:**
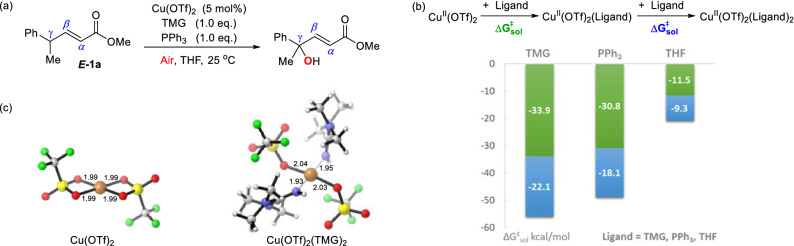
(**a**) The Representative Reaction for the DFT Calculation. (**b**) Binding of TMG, PPh_3_, and THF with Cu^II^(OTf)_2_. Binding Free Energies Are in kcal/mol. (**c**) The Structures of Cu^II^(OTf)_2_ and Cu^II^(OTf)_2_(TMG)_2_. Bond Distances Are Given in Angstroms (Å). *Note* CYLview, 1.0b, https://www.cylview.org/download.html.

The Cu^II^(OTf)_2 _catalyzed reaction of methyl-(*E*)-4-phenylpent-2-enoate ***E-1a*** with 1-equivalent TMG and 1-equivalent PPh_3_ was selected as a representative case for the DFT calculations (Scheme 3a). We first examined the resting state of the copper catalyst. The Cu^II^(OTf)_2_ used in the experiment could be coordinated to the TMG base, the reducing agent PPh_3_ or the solvent THF. As shown in Scheme 3b,c, the coordination of one TMG molecule to the Cu^II^(OTf)_2_ complex is exergonic by 33.9 kcal/mol. The subsequent coordination of a second TMG molecule to form the Cu^II^(OTf)_2_(TMG)_2_ complex would release 22.1 kcal/mol of energy. The computational results suggest TMG is a stronger ligand than PPh_3_ or THF. The binding with one molecule of PPh_3_ or THF is less favorable than that of TMG by 3.1 or 22.4 kcal/mol, respectively. In addition, the generation of cationic copper species from the dissociation of an OTf^ɵ^ anion of the corresponding neutral copper catalysts are all endergonic (Scheme S1). Thus, the four-coordinated Cu^II^(OTf)_2_(TMG)_2_ (Scheme 3c) is considered to be the resting state of the copper catalyst in this reaction.

### Reaction mechanism of copper-catalyzed *γ*-hydroxylation

Having determined the resting state of the copper catalyst, we then explored the mechanism of each elementary reaction step (i.e. deprotonation, O_2_ activation, and reduction) of the copper-catalyzed *γ*-hydroxylation of ***E-1a***. For each step (except for O_2_ activation), both reaction pathways with and without the involvement of copper catalyst were computed to examine the role of copper catalyst.

**Figure 1:**
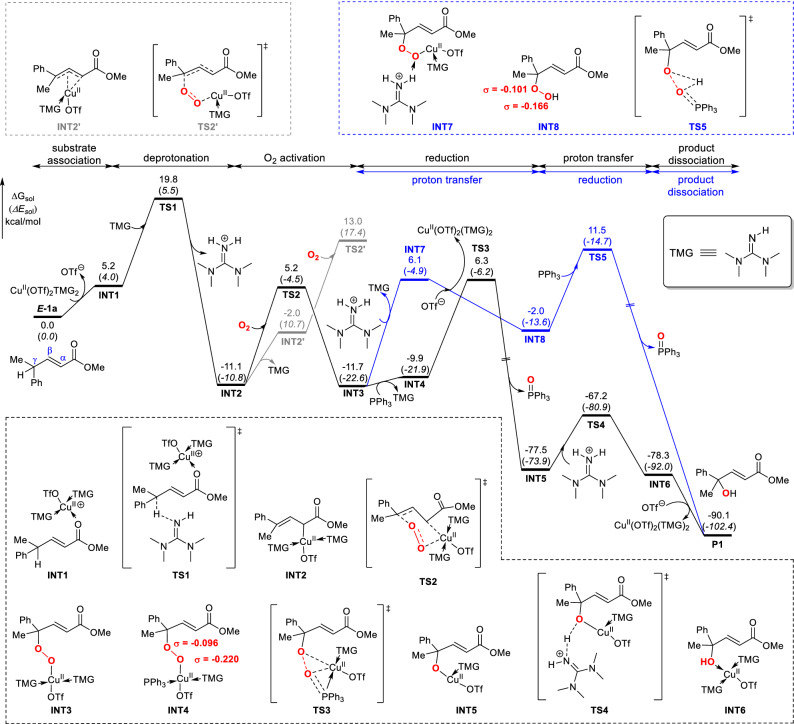
Computed energy profile of copper-catalyzed vinylogous aerobic oxidation of *α*,*β*-unsaturated ester. Relative free energies (electronic energies) are in kcal/mol. The Hirshfeld charges of O atoms (highlighted in red) on **INT4** and **INT8** are listed.

As of the deprotonation step, the direct deprotonation of substrate ***E-1a*** by the base (TMG) via **TS1′** in the absence of copper catalyst requires an energy barrier of 22.3 kcal/mol and this step is energetically uphill by 17.0 kcal/mol, which is thermodynamically unfavorable (Scheme S2). In the copper involved pathway as shown in Fig. 1, the Cu^II^(OTf)_2_(TMG)_2_ catalyst first dissociates an OTf^ɵ^ ligand and binds with ***E-1a***, generating a cationic copper-substrate species **INT1**. Subsequently, TMG abstracts a proton from **INT1** via transition state **TS1** with a free energy barrier of 19.8 kcal/mol and leads to a stable Cu^II^ *σ*-complex, **INT2** (−11.1 kcal/mol). The computational results thus suggest that the coordination of copper with the carbonyl group facilitates the deprotonation of ***E-1a*** and the formation of stable *σ*-complex. Further Hirshfeld population analysis demonstrates the C_*γ*_ hydrogen atoms carries more positive charges when copper catalyst is bound to ***E-1a*** (Scheme S2). This indicates that the polarity-induced effect by the copper catalyst makes the hydrogen more acidic and thus easier to be activated^49–51^. Hence, copper acts as a acid Lewis to mediate the first deprotonation step.

**Figure 2:**
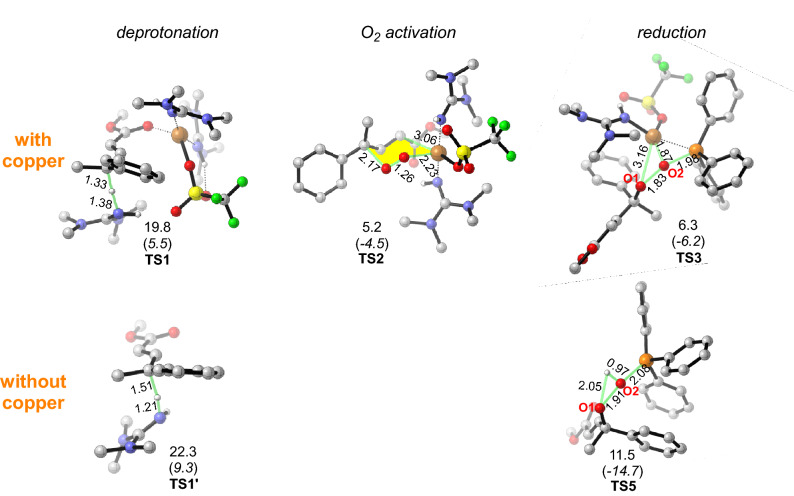
The deprotonation, O_2_ activation and reduction transition states. Relative free energies (electronic energies) are in kcal/mol. The bond distances are given in Angstroms (Å). *Note* CYLview, 1.0b, https://www.cylview.org/download.html.

Following the deprotonation step, the molecular oxygen approaches the Cu^II^ center and attacks the C_*γ*_ of ***E-1a*** substrate in a concerted manner via a six-membered chair-like transition state (**TS2**, Fig. 2) which leads to a peroxide bridge between Cu and substrate^52–55^. The distances of O–Cu and O–C_*γ*_ in **TS2** are 2.23 and 2.17 Å, respectively. Moreover, the O–O bond increases from 1.21 Å in O_2_ to 1.26 Å in **TS2**, which indicates that the O_2_ has been activated. This oxygen activation step needs to overcome a free energy barrier of 16.3 kcal/mol and results in a *γ*-peroxy copper intermediate **INT3**. To understand the regioselectivity, the formation of *α*-hydroxylated product by the oxygen addition at the *α*-carbon via transition state ***α-TS2*** was also calculated (Figure S1). The free energy of ***α-TS2*** is 1.3 kcal/mol higher than that of **TS2** which leads to the *γ*-hydroxylated product, in line with the experimental observation that *γ*-hydroxylated product is more favorable.

To explore the possibility of oxygen activation by binuclear copper species^52,53,56–58^, we computed the reactions of O_2_ with Cu^II^(OTf)_2_(TMG)_2_ and Cu^I^(OTf)(TMG)_3_, respectively, which afford peroxide bimetallic copper compounds (Scheme S3). The former reaction is endergonic with 88.5 kcal/mol and thus can be ruled out. Although the latter one has a relatively low reaction free energy (14.9 kcal/mol), the formation of Cu^I^(OTf)(TMG)_3_ by the disproportionation of Cu^II^(OTf)_2_(TMG)_2_ needs 73.5 kcal/mol of energy which excludes the involvement of Cu^I^. The computational results thus suggest that the oxygen molecule is unlikely to be activated by binuclear copper complexes.

After the O_2_ activation step, the generated *γ*-peroxy copper species **INT3** is reduced to the *γ*-hydroxylated product. The reduction process with and without copper (black and blue path in Fig. 1, respectively) was examined. In the black path, **INT3** first undergoes rapid ligand exchange with PPh_3_ to yield a complex **INT4** which is further reduced by PPh_3_ through transition state **TS3**. In this transition state, PPh_3_ attacks the distal peroxide oxygen while the copper transfers to the proximal oxygen in a concerted way to yield **INT5**. The subsequent proton transfer of **INT5** via **TS4** gives intermediate **INT6** which proceeds ligand exchange to release the *γ*-hydroxylated product and regenerate Cu^II^(OTf)_2_(TMG)_2_ catalyst to complete the catalytic cycle. Alternatively, in the reduction process without the participation of copper (blue path), the **INT3** first abstracts a proton from the protonated TMG and dissociates from copper to yield the hydroperoxyl compound **INT8**. Then **INT8** undergoes reduction with PPh_3_ via a concerted proton shift transition state^59^ (**TS5**) leading to the *γ*-hydroxylation product. The calculated activation barriers for the reduction process with and without the involvement of copper (**TS3** vs. **TS5**) are 18.0 and 23.2 kcal/mol, respectively, which indicates that copper facilitates the reduction step. This could be attributed to two main reasons: (1) the incorporation of copper serves to withdraw electron density from the peroxy which helps to polarize the O–O bond as suggested by a larger difference in charges of O atoms on **INT4** than that for **INT8** (Fig. 1); (2) additional interaction between Cu and the phosphorous atom stabilizes **TS3**.

Overall, the black path of Fig. 1 that involves substrate association, deprotonation, O_2_ activation, reduction, proton transfer and product dissociation was calculated to be the most favorable pathway for the *γ*-hydroxylation of ***E-1a***. The copper-assisted deprotonation step is the rate-determining step, where TMG base plays a pivotal role in hydrogen abstraction. This result coincides with the experimental observation that in the absence of TMG there is no product generated^45^. Furthermore, our computational results suggest the activation of O_2_ molecule proceeds via a six-membered chair-like transition state, which is different from the common end-on or side-on O_2_ activation model^7^ and accounts for the regioselective *γ*-carbon activation. In addition, a copper-mediated reduction process in which copper helps to polarize the O–O bond was unveiled by our computation. The reduction of peroxy complexes by phosphine has been reported in many aerobic reactions^60–65^, while the detailed mechanism is rarely studied. The transition-metal assisted reduction model established in this work provides a possible mechanism for similar reactions. Notably, copper remains as Cu^II^ oxidation state through the whole catalytic cycle and plays vital roles in multiple steps to facilitate the deprotonation, O_2_ activation, as well as reduction.

### The effect of different reducing agents

**Figure 3:**
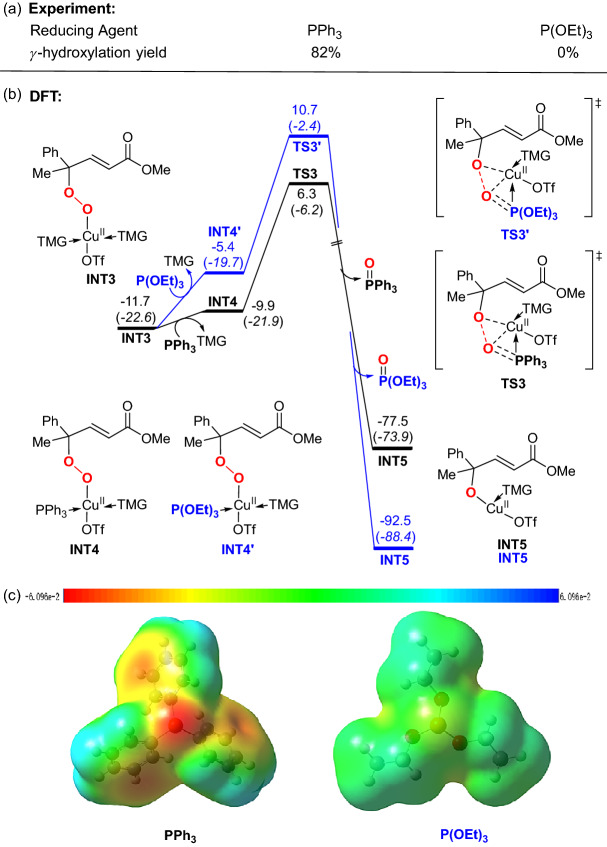
(**a**) The reaction yields of *γ*-hydroxylated productusing different reducing agents. (**b**) Theoretically calculated reduction process using different reducing agents. Relative free energies (electronic energies) are in kcal/mol. (**c**) The electrostatic potential surfaces (ESP) of PPh_3_ and P(O)Et_3_ mapped between − 6.096e−2 to + 6.096e−2. Red as negative extreme and blue as positive extreme. *Note* GaussView6, https://gaussian.com/gv6main/.

The reducing agent plays an important role in this copper-catalyzed vinylogous *γ*-hydroxylation. A high-yield (82%) of the *γ*-hydroxylated product was obtained with PPh_3_ as a reducing agent, however, the reaction did not occur when PPh_3_ was changed to P(OEt)_3_ (Fig. 3a). In line with the experiment, our computation in Fig. 3b shows that the reduction with P(OEt)_3_ (**TS3′**) is disfavored by 4.4 kcal/mol compared to the corresponding reduction process with PPh_3_ (**TS3**), which supports the PPh_3_ is a better reducing agent than P(OEt)_3_ for this reaction. The calculated electrostatic potential surfaces (ESPs)^66–69^ in Fig. 3c clearly indicate the P atom of PPh_3_ is more electron-rich. Therefore, the PPh_3_ could better stabilize the electron-deficient Cu center than P(OEt)_3_, accounting for the lower barrier of **TS3**.

### The reactivity of different substrates

**Figure 4:**
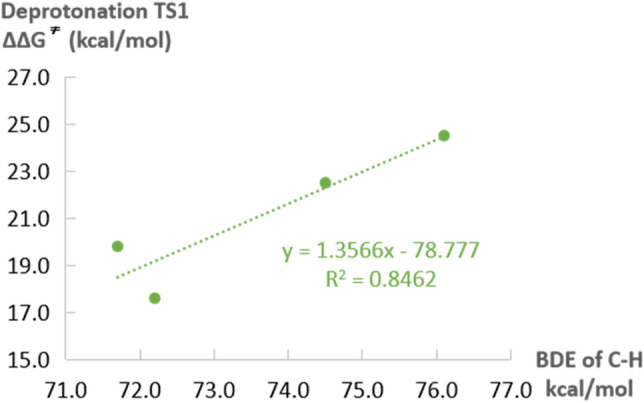
The relationship between deprotonation the energy barriers and the corresponding C–H bond dissociation energy (BDE) for different substrates.

The original experimental work reported that *γ*-aryl-*γ*-alky-disubstituted *α*,*β*-unsaturated compound ***E-1a*** and *γ*-aryl-*γ*-alky-disubstituted *β*,*γ*-unsaturated compound ***E-1b*** are both reactive and generates the same *γ*-hydroxylated product (Table 1, entry 1 and 2). The *γ*,*γ*-dialkyl-substituted *β*,*γ*-unsaturated substrate 2b also exhibits good reactivity (entry 3). However, the *γ*,*γ*-dialkyl-substituted *α*,*β*-unsaturated compound ***E-2a*** was completely inert (entry 4). Further computation was performed to understand the observed reactivities of different *α*,*β*- or *β*,*γ*-unsaturated substrates. As indicated by our DFT studies, the deprotonation process is the rate-determining step. Thus, we examined the deprotonation transition states for different substrates to evaluate their reactivities. The calculated energy barriers of deprotonation follow the order: ∆∆G^≠^(**TS1**_***E-1b***_) < ∆∆G^≠^(**TS1**_***E-1a***_) < ∆∆G^≠^(**TS1**_***2b***_) < ∆∆G^≠^(**TS1**_***E-2a***_), which is consistent with the trend of yield (Table 1). To obtain deep insights, the bond dissociation energy (BDE)^70^ of the corresponding C–H bonds (highlighted in red) were calculated to evaluate the intrinsic acidity. As shown in Figs. 4, a good linear association between the deprotonation free energy barriers (∆∆G^≠^) and the BDE values were observed. This indicates that the inherent strength of the C–H bond is a key factor affecting the deprotonation process, which is related to the substrate reactivity. In addition, the (*E*)-*α*,*β*-unsaturated esters ***E-1a*** and (*E*)-*β*,*γ*-unsaturated esters ***E-1b*** generated the same *γ*-hydroxylated product in experiments. Based on the understanding of reaction mechanism and computational results, we concluded that the deprotonation of both (*E*)-*α*,*β*-unsaturated ester and (*E*)-*β*,*γ*-unsaturated ester would generate the same Cu^II^ *σ*-complex and thus leads to the same *γ*-hydroxylated isomer. However, the reactivities of these two substrates (***E-1a*** and ***E-1b***) are different. ***E-1a*** has a higher deprotonation energy barrier than ***E-1b*** by 2.2 kcal/mol corresponding with a lower yield of ***E-1a*** and further supports the deprotonation is the rate-determining step.

**Table 1:**
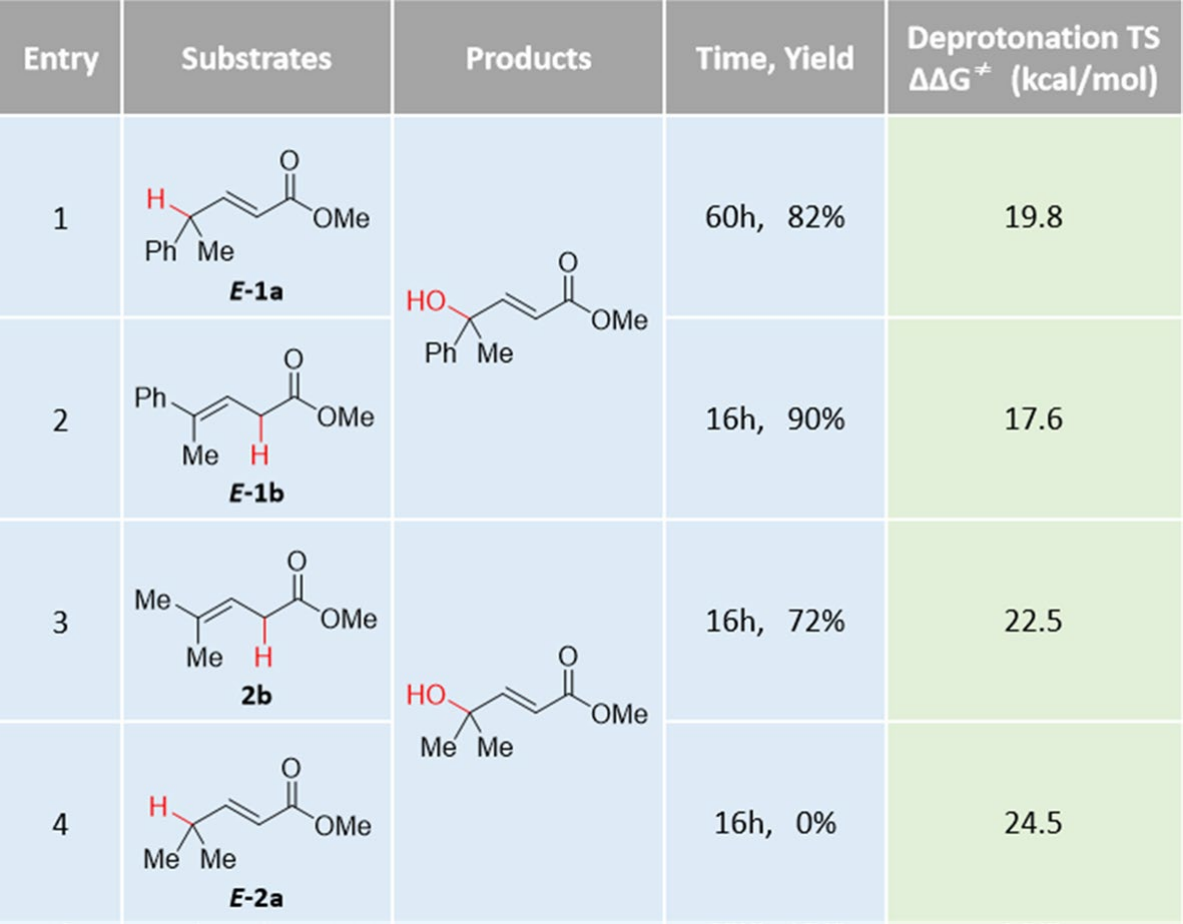
The reaction yields and the calculated energy barriers of deprotonation transition states for different substrates.

### The mechanism of byproduct generation

One of the main problems for the hydroxylation of *α*,*β*-unsaturated compounds is the formation of oxidative fragments. In the original experiment, a side product acetophenone was detected and proposed to be generated by oxidative fragmentation of a four-membered endoperoxide intermediate **D** as depicted in Scheme 2. We also explored the formation of acetophenone byproduct. As shown in Scheme 2, the main product path and the byproduct path differentiate from the *γ*-peroxy copper intermediate.

**Figure 5:**
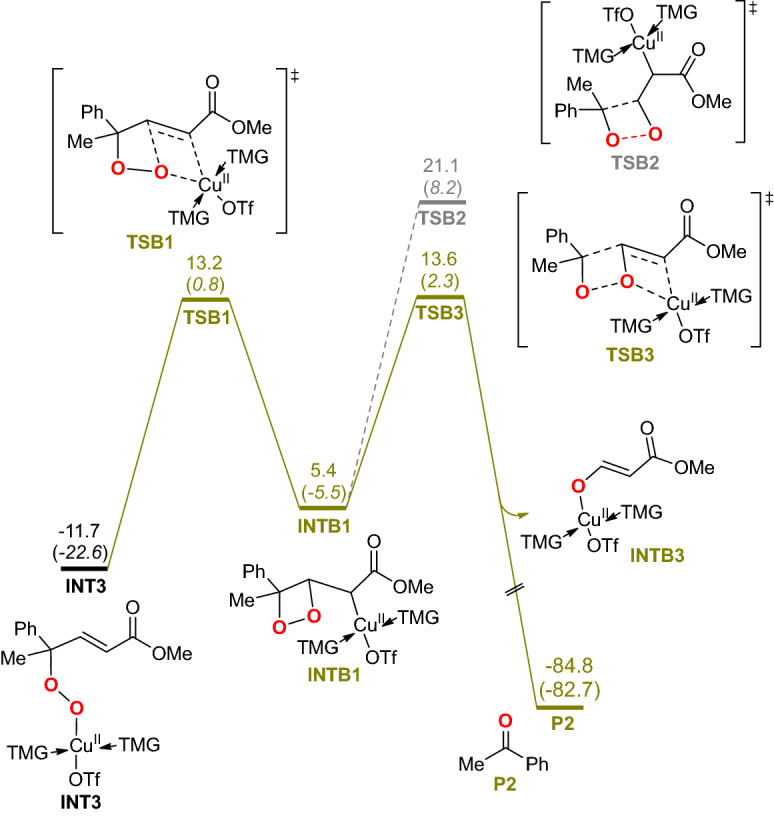
The possible formation routes of acetophenone byproduct. Relative free energies (electronic energies) are in kcal/mol.

Starting with this intermediate (**INT3)**, three possible pathways leading to the acetophenone byproduct were examined (Scheme S4). A reaction pathway involving a peroxide radical^71–78^ which is formed via the homolysis of Cu–O bond of **INT3** needs to overcome a reaction energy barrier of 29.2 kcal/mol (path I in Scheme S4). Alternatively, **INT3** could undergo an intramolecular alkene insertion into the Cu–O bond via **TSB1** to form four-membered endoperoxide **INTB1** which is similar to the endoperoxide intermediate **D** proposed in the original experimental work (Fig. 5). But the further homolysis of the C–C and O–O bond of **INTB1** via **TSB2** to cleavage the four-membered ring which affords the byproduct is highly unfavorable with an energy barrier of 32.8 kcal/mol. Instead, our computational results demonstrated that copper can facilitate the cycloelimination process by transferring to the *β*-oxygen atom of endoperoxide (**TSB3**) to maintain the conjugated *α*,*β*-unsaturated structure, lowering the activation barrier by 7.5 kcal/mol. The direct cycloelimination of four-membered peroxide complexes was proposed in many works^79–83^ and our results suggested a possible role of transition metal to facilitate the oxidative fragmentation process and to provide the theoretical basis for further reaction improvement.

## Conclusion

**Figure 6:**
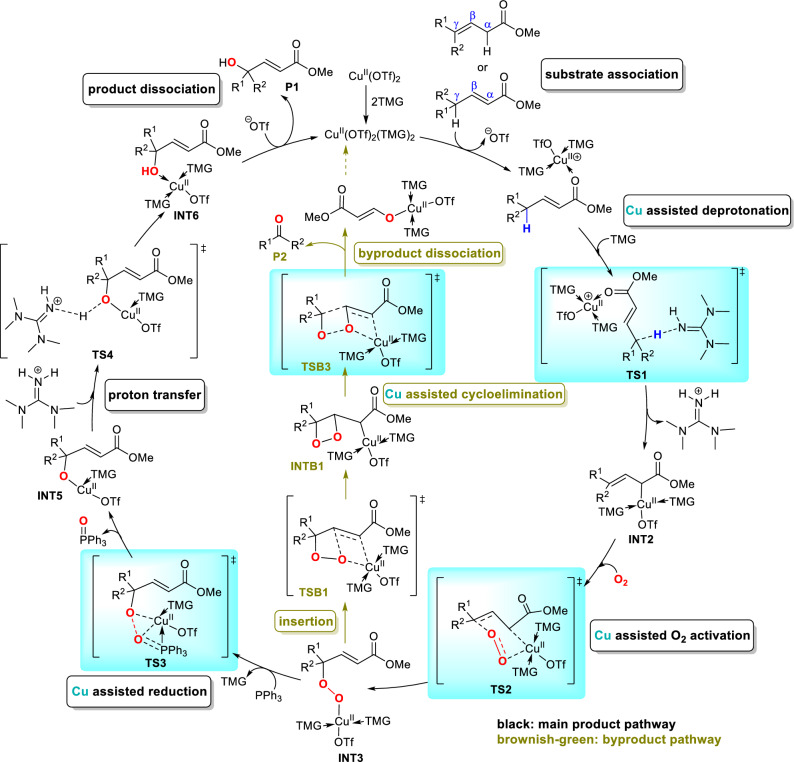
The proposed catalytic cycle for the copper-catalyzed vinylogous aerobic oxidation of *α*,*β*- and *β*,*γ*-unsaturated compounds.

In summary, we have conducted DFT studies on the Cu-catalyzed vinylogous aerobic oxidation of *γ*,*γ*-disubstituted *α*,*β*- and *β*,*γ*-unsaturated compounds. As summarized in Fig. 6, computational results unveiled a detailed reaction mechanism of *γ*-hydroxylation reaction that includes six steps: substrate association, deprotonation, O_2_ activation, reduction, proton transfer and product dissociation (black path), and the deprotonation is the rate-determining step. The regioselectivity is controlled by the O_2_ activation step which prefers to proceed via a six-membered chair-like transition state, leading to a *γ*-oxidation intermediate. Besides, the inefficiency of P(OEt)_3_ and inertness of *γ*,*γ*-dialkyl substituted *β*,*γ*-unsaturated ester were also understood by computations. A pathway consisting of intramolecular alkene insertion, cycloelimination, and product dissociation (brownish-green path) was revealed to account for the acetophenone byproduct generation. The understanding of the reaction mechanism laid a theoretical foundation for further reaction development.

The copper retains the + 2 oxidation state (Cu^II^) and participates in the whole catalytic cycle of both main product and byproduct formation. Notably, it plays vital roles in multiple steps: (1) facilitates the substrate deprotonation by increasing the acidity of C–H; (2) activates O_2_ via a six-membered chair-like model which is different from the common end-on or side-on O_2_ activation model; (3) assists the reduction of peroxyl intermediate through a 1,2-migration transition state; and (4) promotes the cycloelimination of endoperoxide by transferring to oxygen to maintain the conjugated *α*,*β*-unsaturated structure. The understanding of mechanism for O_2_ activation and O–O bond cleavage are essential for the development of transition metal-catalyzed aerobic reactions. The six-membered chair-like transition state for O_2_ activation and copper-mediated O–O bond cleavage models (**TS3** and **TSB3**) have not been documented in literature to the best of our knowledge and may provide hints for the mechanistic studies and future development of transition metal-catalyzed aerobic oxidation reactions.

## Computational details

All the calculations were performed with Gaussian 09 package^84^. Geometries were optimized in gas phase by using unrestricted B3LYP-D3^85–88^ and a mixed basis set of SDD^89,90^ for Cu and P, and 6-31G(d)^91,92^ basis set for all other atoms. Optimized geometries were verified by frequency computations as minima (zero imaginary frequencies) or transition state (a single imaginary frequency) at the same level of theory. The transition states (TSs) were also confirmed by viewing normal mode vibrational vector. Solvent effect was included by single-point energy calculation using SMD model with tetrahydrofuran (THF) as the solvent and B3LYP-D3 method with def2-TZVP basis set for Cu and P, and 6-311+G(d, p) basis set for other atoms^93–97^. All relative Gibbs free energies and electronic energies (at 298.15 K and 1 atm) were reported in kcal/mol. The Hirshfeld charges^98^ were obtained from the B3LYP-D3 single-point calculation. The 3D structures were generated by CYLview^99^.

## Supplementary information

Supplementary Informations.
