# Supplementary material for: Computational exploration of copper catalyzed vinylogous aerobic oxidation of unsaturated compounds
Source: Sci Rep. 2021 Jan 14;11:1304. doi: 10.1038/s41598-020-80188-2 (PMC7809353; doi:10.1038/s41598-020-80188-2)
Supplement: Supplementary file 1 — Supplementary Informations. [file 41598_2020_80188_MOESM1_ESM.pdf]

Supporting Information

Computational Exploration of Copper Catalyzed Vinylogous Aerobic Oxidation of Unsaturated Compounds

Ting Wang,[a] Yu Zhou,[a,b] Yao Xu,[a,b] Gui-Juan Cheng*[a]

[a] Warshel Institute for Computational Biology, Shenzhen Key Laboratory of Steroid Drug Development, School of Life and Health Sciences, The Chinese University of Hong Kong (Shenzhen), Shenzhen 518172, China. *E-mail: chengguijuan@cuhk.edu.cn

[b] School of Life Sciences , University of Science and Technology of China , 230027 Hefei , Anhui , China.

Computational Results

Scheme S1. The Active Copper Catalyst. Relative Free Energies Are in kcal/mol Calculated in THF Solvent.

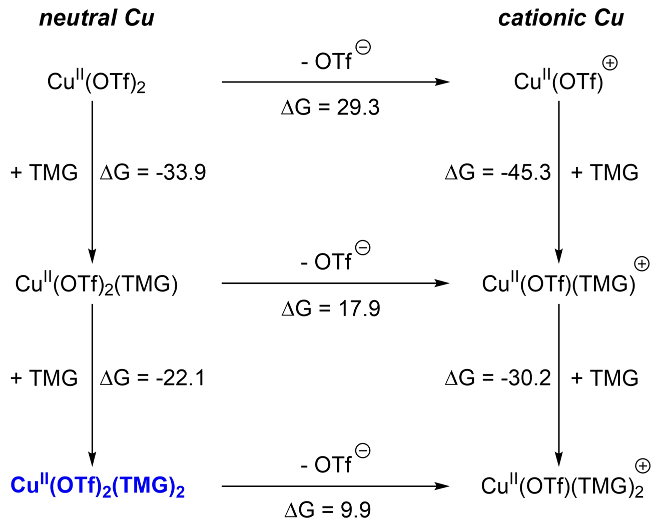

Scheme S2. The Depronation of *E*-1a by TMG with or without the coordination of Cu-catalyst. Relative free energies (electronic energies) are in kcal/mol.

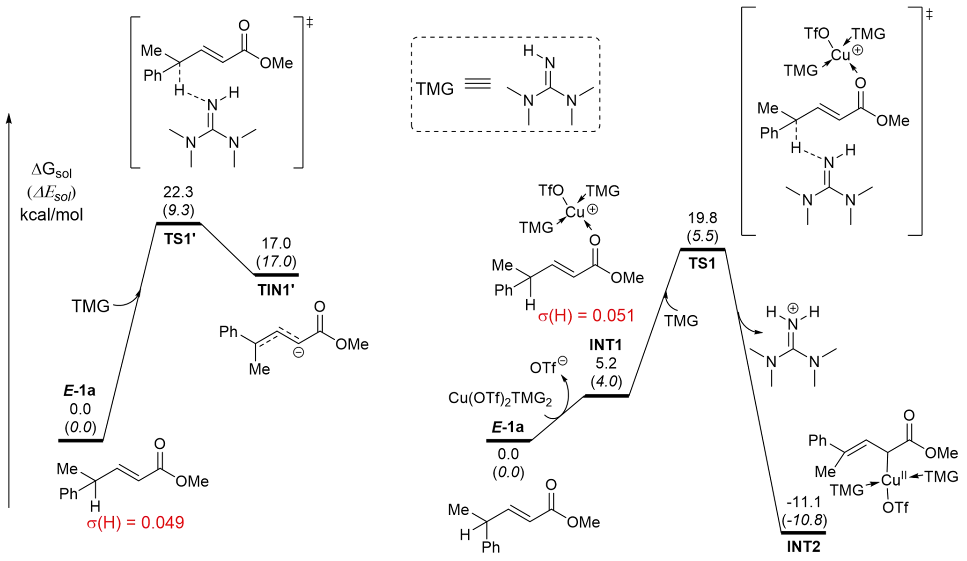

Scheme S3. The Reaction of $Cu^{II}(OTf)_2(TMG)_2$ and $Cu^{I}(OTf)(TMG)_3$ with O_2 and the Disproportionation of $Cu^{II}(OTf)_2(TMG)_2$.

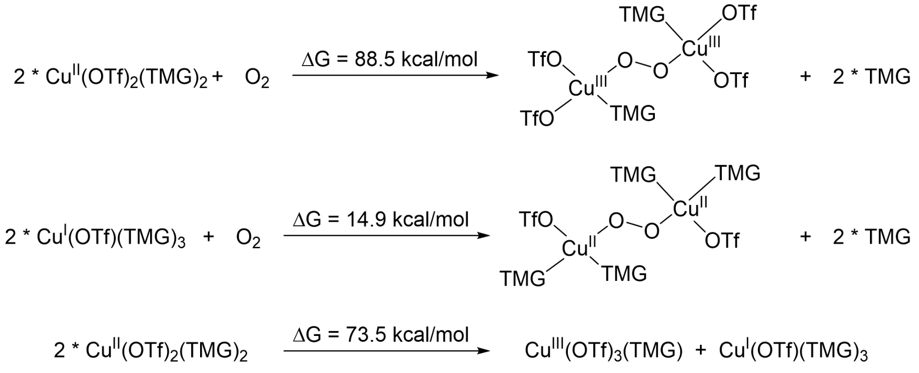

Scheme S4. Computed Reaction Routes and Energies for the Formation of Acetophenone Product. Relative Free Energies (Electronic Energies) are in kcal/mol.

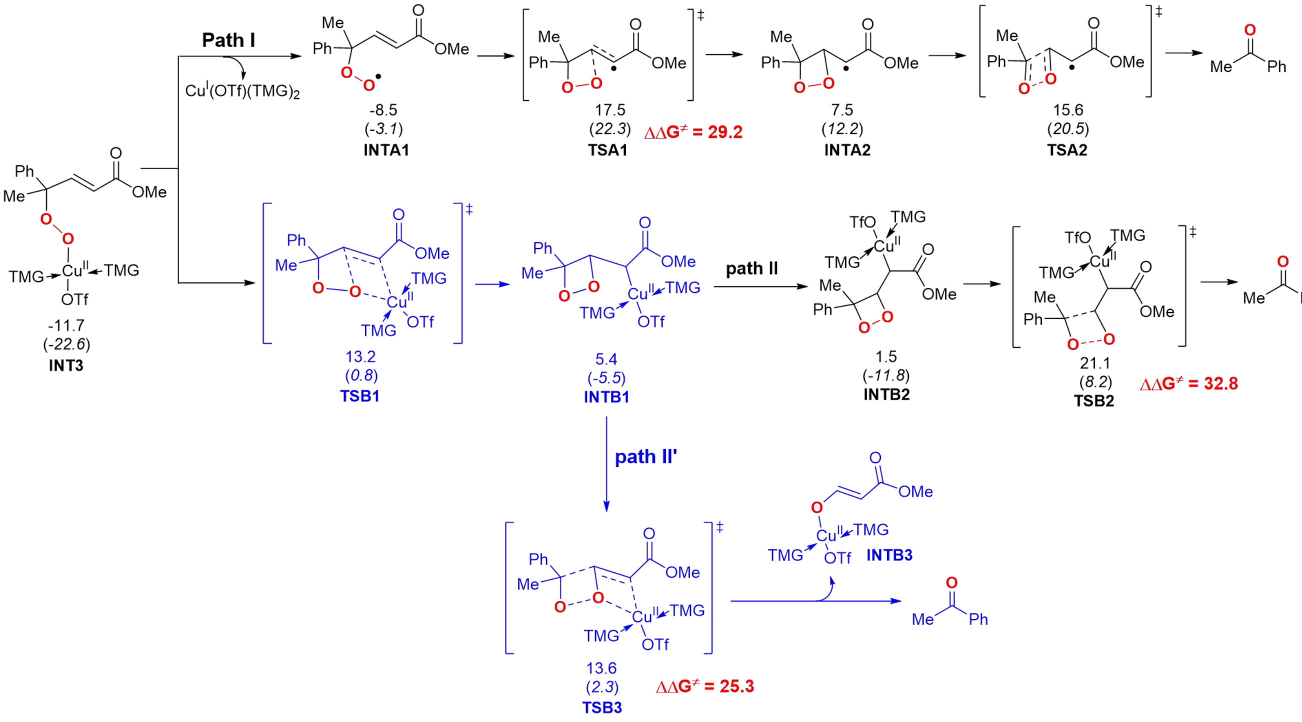

Figure S1. The transition state of O_2 addition of *α*-carbon. Relative free energies (electronic energies) are in kcal/mol. The bond distances are given in angstroms. Note: CYLview, 1.0b, https://www.cylview.org/download.html.

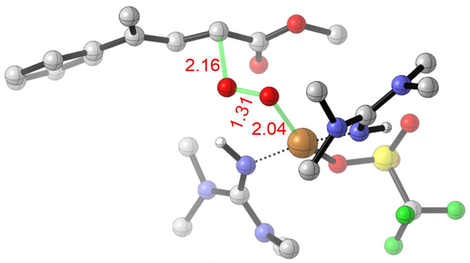

6.5
(-3.4)
α-**TS2**

Table S1. Corrections to zero point energies, enthalpies, free energies and electronic potential energies (in Hartree) and imaginary frequencies (IF) (cm^{-1}) of optimized structures which were calculated at uB3LYP-D3/6-311++G(d,p)+def2-TZVP(Cu and P)//uB3LYP-D3/6-31G(d)+SDD(Cu and P) level in solvent (Tetrahydrofuran) at 298.15 K and 1 atm.

Geometry	$cZPE_{298,gas}$	$cH_{298,gas}$	$cG_{298,gas}$	$E_{0,sol}$	IF
***E*-1a**	0.23458	0.24934	0.192108	-616.362400	1
***E*-1b**	0.233973	0.24896	0.190256	-616.362378	1
***E*-2a**	0.181587	0.193208	0.145052	-424.569659	1
**2b**	0.180493	0.192532	0.142015	-424.567334	1
**O_2**	0.003775	0.007082	-0.016204	-150.377732	1
**$OTf^{\ominus}$**	0.027239	0.035368	-0.005301	-961.805528	1
**THF**	0.117444	0.123306	0.088966	-232.5329994	1
**TMG**	0.189610	0.200452	0.155375	-362.730975	1
**$(TMG+H)^{\oplus}$**	0.203580	0.214869	0.169383	-363.208123	1
**PPh_3**	0.274541	0.291354	0.228213	-1036.548211	1
**$P(O)Ph_3$**	0.279582	0.297199	0.233100	-1111.832815	1
**$P(OEt)_3$**	0.214029	0.229219	0.169686	-804.948639	1
**$P(O)(OEt)_3$**	0.219508	0.235356	0.174555	-880.2717638	1
**$Cu^{II}(OTf)^{\oplus}$**	0.027907	0.037905	-0.009117	-2602.012244	1
**$Cu^{II}(OTf)_2$**	0.057730	0.076075	0.007857	-3563.886732	1
**$Cu^{II}(OTf) (TMG)^{\oplus}$**	0.221616	0.242670	0.169058	-2964.838177	1
**$Cu^{II}(OTf)(TMG)_2{}^{\oplus}$**	0.414469	0.446781	0.349629	-3327.642405	1
**$Cu^{II}(OTf)_2(TMG)$**	0.250777	0.280415	0.188324	-3926.696795	1
**$Cu^{II}(OTf)_2(TMG)_2$**	0.443628	0.484277	0.370482	-4289.489789	1
**$Cu^{II}(OTf)_2(THF)$**	0.178199	0.20264	0.120446	-3796.461701	1
**$Cu^{II}(OTf)_2(THF)_2$**	0.298789	0.329222	0.234834	-4029.034998	1
**$Cu^{II}(OTf)_2(PPh_3)_2$**	0.33392	0.370306	0.258199	-4600.510931	1
**$Cu^{II}(OTf)_2(PPh_3)_2$**	0.61085	0.664564	0.51521	-5637.121456	1
**$Cu^{III}(OTf)_3(TMG)$**	0.279597	0.317717	0.206221	-4888.285995	1
**$Cu^{I}(OTf)(TMG)_3$**	0.604338	0.648855	0.524609	-3690.566328	1
**Cu^{III}-dimer**	0.510737	0.572049	0.411554	-8003.751891	1
**Cu^{II}-dimer**	0.834593	0.902409	0.727096	-6806.029473	1
**INT1**	0.653005	0.699983	0.57187	-3944.042352	1
**TS1**	0.839265	0.897373	0.744741	-4306.767577	-1299.08
**INT2**	0.637363	0.685122	0.553813	-3943.587127	1
**INT2'**	0.445361	0.481349	0.376203	-3580.819453	1
**TS2**	0.6424	0.692535	0.554654	-4093.955971	-265.82
**TS2'**	0.450189	0.489189	0.374342	-3731.187596	-244.85
***α*-TS2**	0.642741	0.692566	0.555381	-4093.954607	-295.12

Geometry	$cZPE_{298,gas}$	$cH_{298,gas}$	$cG_{298,gas}$	$E_{0,sol}$	IF
**INT3**	0.646473	0.695541	0.560759	-4093.988961	1
**INT4**	0.730031	0.785457	0.633828	-4767.808216	1
**INT4'**	0.670746	0.723763	0.58034	-4536.201816	1
**TS3**	0.727753	0.783205	0.632204	-4767.780889	-558.47
**TS3'**	0.668565	0.721444	0.576257	-4536.172067	-623.10
**INT5**	0.448856	0.486215	0.374051	-3656.037113	1
**TS4**	0.65212	0.699695	0.570605	-4019.256028	-1046.12
**INT6**	0.656709	0.704613	0.575172	-4019.278307	1
**INT7**	0.659938	0.709247	0.57448	-4094.437453	1
**INT8**	0.24194	0.258807	0.197878	-766.7653116	1
**TS5**	0.515722	0.549745	0.448515	-1803.319152	-286.67
**INTA1**	0.229693	0.246423	0.18396	-766.123026	1
**TSA1**	0.228077	0.244144	0.183487	-766.0810124	-756.32
**INTA2**	0.229399	0.245655	0.184757	-766.098314	1
**TSA2**	0.22711	0.243376	0.182252	-766.082895	-764.23
**TSB1**	0.645867	0.694138	0.562439	-4093.951	-436.26
**INTB1**	0.647345	0.696036	0.561648	-4093.962609	1
**INTB2**	0.648621	0.696858	0.566675	-4093.973842	1
**TSB2**	0.64417	0.692788	0.561669	-4093.937594	-346.52
**TSB3**	0.644673	0.69349	0.559466	-4093.947411	-526.88
**INTB3**	0.506067	0.546371	0.432022	-3709.056578	1
**P1**	0.238602	0.254315	0.195555	-691.608986	1
**P2**	0.138741	0.147393	0.106230	-385.026416	1

Table S2. The bond dissociation energy (BDE) of the corresponding C-H bonds on different substrates were calculated at/uB3LYP-D3/6-31G(d)+SDD(Cu and P) level in gas phase at 298.15 K and 1 atm.

Geometry	$cH_{298,gas}$	$E_{0,gas}$	$H_{298,sol}$	IF
***E*-1a**	0.24934	-616.161892	-615.912552	1
***E*-1b**	0.24896	-616.162359	-615.913399	1
***E*-2a**	0.193208	-424.424911	-424.231703	1
**2b**	0.192532	-424.42166	-424.229128	1
**H_radical**	0.00236	-0.500273	-0.497912	1
***E*-1a_radical**	0.236337	-615.536762	-615.300425	1
***E*-1b_radical**	0.236337	-615.536761<br>6	-615.300424<br>6	1
***E*-2a_radical**	0.179526	-423.792023	-423.612496	1
**2b_radical**	0.179527	-423.792023	-423.612496	1

Table S3. Cartesian coordinates (in Å) of related structures which were calculated at the uB3LYP-D3/6-31G(d)+SDD(Cu an P) level of theory.

***E*-1a**

C	2.885749	-0.186822	0.156522
O	3.884429	-0.428568	-0.729128
O	2.981494	-0.413809	1.348021
C	5.079436	-0.981288	-0.159204
H	5.770811	-1.111989	-0.992775
H	5.500966	-0.301977	0.587899
H	4.869484	-1.941758	0.321097
C	1.703350	0.378415	-0.530135
H	1.776871	0.528580	-1.603816
C	0.597550	0.682962	0.159026
H	0.602359	0.497430	1.232976
C	-0.660647	1.267276	-0.427188
C	-1.838648	0.321472	-0.206423
C	-2.288637	0.011863	1.085467
C	-2.491632	-0.262186	-1.298249
C	-3.365468	-0.853423	1.278397
H	-1.794277	0.448429	1.949685
C	-3.572467	-1.126315	-1.109806
H	-2.151249	-0.036987	-2.306545
C	-4.012886	-1.425240	0.180283
H	-3.698803	-1.081937	2.287317
H	-4.067782	-1.566208	-1.971491
H	-4.852363	-2.098706	0.330181
C	-0.930505	2.667897	0.167157
H	-1.849051	3.089072	-0.255238
H	-1.050292	2.620408	1.255059
H	-0.098300	3.345689	-0.051491
H	-0.516916	1.381315	-1.509084

***E*-1b**

C	-0.604096	0.914753	0.077249
C	0.523570	0.257812	-0.249842
H	0.461949	-0.778093	-0.570998
C	-0.622142	2.380436	0.455585
H	-0.966953	3.001865	-0.382187
H	-1.306545	2.562747	1.291745
H	0.367556	2.748725	0.738206
C	-1.918304	0.216015	0.043382
C	-2.027636	-1.152786	0.352573
C	-3.097017	0.909151	-0.285556
C	-3.257695	-1.805646	0.306662
H	-1.141353	-1.701785	0.656660
C	-4.329364	0.256470	-0.331741
H	-3.052462	1.967409	-0.524842
C	-4.416362	-1.105205	-0.038664
H	-3.313468	-2.862338	0.555338
H	-5.222826	0.815076	-0.598902
H	-5.376468	-1.613268	-0.068989
C	1.917825	0.820181	-0.250918
H	2.128531	1.370951	-1.181814
H	2.091426	1.543903	0.553282
C	2.982273	-0.260591	-0.146164
O	4.195108	0.295255	0.071689
O	2.804244	-1.453798	-0.259281
C	5.288756	-0.634383	0.152500
H	6.178562	-0.026899	0.321655
H	5.137668	-1.334780	0.979019
H	5.380725	-1.202417	-0.777825

***E*-2a**

C	-1.506270	-0.000539	0.156707
O	-2.516010	0.003527	-0.749583
O	-1.690665	-0.006101	1.359410
C	-3.835661	0.000567	-0.186962
H	-4.519152	0.003510	-1.037131
H	-3.991002	-0.890481	0.428863
H	-3.992081	0.886655	0.435730
C	-0.192663	0.002619	-0.524117
H	-0.197491	0.007754	-1.610822
C	0.943650	-0.000996	0.183286
H	0.862106	-0.006146	1.271578
C	2.332965	0.001535	-0.387269
C	3.089535	-1.268403	0.049383
H	4.104124	-1.272894	-0.365918
H	3.173143	-1.314948	1.142389
H	2.574708	-2.173990	-0.288827
H	2.263888	0.005967	-1.483814

C	3.089174	1.268051	0.059543
H	4.103670	1.276226	-0.355851
H	2.574038	2.176218	-0.271078
H	3.173065	1.305716	1.152830

2b

C	-2.281393	-0.004053	-0.020779
C	-1.046856	-0.402127	0.315520
H	-0.853657	-1.465991	0.436363
C	-3.389575	-1.010686	-0.221233
H	-3.045372	-2.037891	-0.065460
H	-3.804296	-0.939605	-1.236752
H	-4.223797	-0.821111	0.469201
C	0.154470	0.471504	0.556947
H	0.269460	0.692347	1.631169
H	0.090472	1.445037	0.062632
C	1.452333	-0.198149	0.133323
O	2.436316	0.719744	-0.005529
O	1.620656	-1.386807	-0.030889
C	3.724952	0.185882	-0.352534
H	4.390599	1.047015	-0.422999
H	3.677151	-0.343421	-1.308606
H	4.074240	-0.509203	0.416737
C	-2.701573	1.431758	-0.221524
H	-3.144291	1.569074	-1.217774
H	-1.881294	2.146289	-0.115541
H	-3.479145	1.709184	0.504059

O_2

O	0.000000	0.000000	0.607109
O	0.000000	0.000000	-0.607109

$OTf^{\ominus}$

S	0.925888	-0.000261	0.000003
O	1.240810	-1.447151	0.014835
O	1.241397	0.736127	1.245727
O	1.241403	0.710435	-1.260553
C	-0.940169	0.000354	-0.000005
F	-1.442177	1.255727	-0.012837
F	-1.443471	-0.638601	-1.080790
F	-1.443472	-0.616374	1.093617

THF

C	-1.164041	-0.430082	0.134807
O	0.000179	-1.251689	-0.000611
C	1.164384	-0.429666	-0.134023
C	0.731762	0.996290	0.231837
C	-0.732333	0.995775	-0.232190
H	-1.952417	-0.824615	-0.517231
H	-1.527008	-0.475367	1.173882
H	1.951864	-0.823589	0.519499
H	1.529112	-0.475304	-1.172438
H	0.785616	1.145728	1.317103
H	1.345156	1.764486	-0.249264
H	-1.346061	1.764063	0.248347
H	-0.786320	1.144209	-1.317584

TMG

N	0.092549	1.827548	0.016336
H	-0.826363	2.258436	0.114702
C	0.011216	0.543636	0.010663
N	1.175620	-0.225468	0.117772
N	-1.164416	-0.228848	-0.109043
C	1.386757	-1.310185	-0.838835
H	0.435608	-1.770143	-1.106500
H	1.864742	-0.944348	-1.763262
H	2.038805	-2.072591	-0.396696
C	2.397033	0.502993	0.434443
H	2.774539	1.078230	-0.424760
H	2.207751	1.204290	1.248004
H	3.162199	-0.218797	0.742455
C	-2.371906	0.470991	-0.509415
H	-2.156733	1.132035	-1.353497
H	-3.118522	-0.264678	-0.827441
H	-2.817637	1.070860	0.304605
C	-1.404088	-1.278115	0.881093
H	-1.926142	-0.884800	1.770002
H	-2.023171	-2.070939	0.444410
H	-0.455419	-1.706097	1.204839

$(TMG+H)^{\oplus}$

N	0.000329	1.847524	0.000357
H	0.793046	2.372935	-0.339840
C	0.000059	0.498995	0.000027

N	-1.174008	-0.158028	0.030180
N	1.174019	-0.158222	-0.030276
C	-1.336060	-1.446030	0.721933
H	-0.438502	-1.687002	1.290803
H	-2.174690	-1.359315	1.419911
H	-1.550162	-2.250893	0.012152
C	-2.421406	0.478540	-0.414172
H	-2.954184	0.945722	0.423527
H	-2.214347	1.222456	-1.185563
H	-3.065250	-0.292839	-0.844190
C	2.421486	0.478383	0.413793
H	2.214555	1.221772	1.185737
H	3.065752	-0.293117	0.842930
H	2.953762	0.946278	-0.423848
C	1.335689	-1.446460	-0.721689
H	2.174088	-1.360099	-1.419986
H	1.549896	-2.251103	-0.011692
H	0.437893	-1.687471	-1.290159
H	-0.792842	2.373194	0.339039

PPh_3

P	-0.000282	-0.001290	-1.260557
C	0.403822	1.607130	-0.436546
C	-1.595741	-0.454002	-0.436902
C	1.190368	-1.155260	-0.437067
C	1.187175	1.727037	0.721768
C	-2.088772	0.161836	0.724068
C	0.902816	-1.896880	0.719321
C	-0.114095	2.773959	-1.023709
C	-2.350192	-1.483100	-1.025188
C	2.461038	-1.285169	-1.022507
C	1.440397	2.982127	1.280323
C	-3.302396	-0.245568	1.282868
C	1.865472	-2.741368	1.277609
C	0.129023	4.026238	-0.458817
C	-3.556381	-1.898029	-0.460032
C	3.425820	-2.120242	-0.458661
C	0.909823	4.133300	0.694808
C	-4.037370	-1.277249	0.695323
C	3.129061	-2.852669	0.693428
H	1.600024	0.837866	1.188316
H	-1.523739	0.962031	1.192401
H	-0.074286	-1.814032	1.185391
H	-0.711448	2.699879	-1.929741
H	-1.988646	-1.962292	-1.932365
H	2.695002	-0.728521	-1.927333
H	2.051191	3.058903	2.176364
H	-3.671790	0.243285	2.180926
H	1.627518	-3.310485	2.172789
H	-0.282800	4.918331	-0.923587
H	-4.124703	-2.698761	-0.926172
H	4.405031	-2.205473	-0.922689
H	1.108168	5.108757	1.131089
H	-4.981100	-1.593008	1.132260
H	3.876588	-3.509693	1.130164

$P(O)Ph_3$

P	-0.000129	-0.001606	0.940429
C	-1.433881	-0.884245	0.230364
C	-0.048686	1.681663	0.230769
C	1.481741	-0.800369	0.229581
C	-1.433111	-1.466457	-1.045611
C	-0.553996	1.972434	-1.044804
C	1.987309	-0.505240	-1.044891
C	-2.587733	-0.955690	1.024203
C	0.464765	2.716911	1.025268
C	2.121294	-1.763981	1.022747
C	-2.582425	-2.093698	-1.529540
C	-0.523766	3.281629	-1.528104
C	3.107587	-1.183573	-1.527969
C	-3.733446	-1.586618	0.538498
C	0.489630	4.024947	0.540364
C	3.243047	-2.437228	0.537982
C	-3.733354	-2.151219	-0.739569
C	0.000623	4.307349	-0.737617
C	3.733711	-2.151501	-0.738657
H	-0.533275	-1.446728	-1.654274
H	-0.986492	1.183532	-1.654012
H	1.520313	0.264753	-1.652799
H	-2.568679	-0.528453	2.022601
H	0.825016	2.486621	2.023697
H	1.740731	-1.963558	2.020260
H	-2.576364	-2.545231	-2.518034
H	-0.917934	3.502141	-2.516560

H	3.497024	-0.949676	-2.515220
H	-4.624373	-1.641908	1.158214
H	0.885945	4.824254	1.160762
H	3.736954	-3.180971	1.157462
H	-4.625988	-2.643403	-1.116564
H	0.019547	5.326711	-1.114051
H	4.608283	-2.675652	-1.114867
O	0.000671	-0.002378	2.443608

$P(OEt)_3$

P	-0.034735	0.004380	0.744223
O	0.176989	-1.456517	-0.107833
O	1.157613	0.897382	-0.083415
O	-1.371526	0.568092	-0.150102
C	1.315496	-2.249911	0.257222
H	2.232675	-1.753692	-0.087309
H	1.381981	-2.355057	1.352221
C	1.155924	-3.610477	-0.400013
H	2.014599	-4.251391	-0.168778
H	0.244309	-4.101519	-0.044233
H	1.084430	-3.497988	-1.486471
C	-2.645719	-0.006837	0.173760
H	-2.668643	-1.055747	-0.151477
H	-2.815082	0.011119	1.262485
C	-3.709779	0.805180	-0.545107
H	-3.531673	0.787239	-1.624942
H	-4.706176	0.393855	-0.346201
H	-3.685873	1.847201	-0.209835
C	1.299033	2.275708	0.289384
H	0.444925	2.848535	-0.096454
H	1.307030	2.384179	1.385996
C	2.602666	2.781305	-0.305122
H	2.590872	2.664944	-1.393445
H	2.747480	3.841380	-0.066702
H	3.449375	2.212288	0.092530

$P(O)(OEt)_3$

P	0.001729	-0.000848	0.354589
O	-1.051359	-1.031734	-0.383565
O	1.423171	-0.393692	-0.381677
O	-0.366846	1.429318	-0.377074
C	-1.091659	-2.395335	0.112902
H	-0.146366	-2.890383	-0.141917
H	-1.195227	-2.378096	1.203773
C	-2.271387	-3.076537	-0.553146
H	-2.339559	-4.118555	-0.220923
H	-3.203873	-2.564951	-0.294563
H	-2.157853	-3.062467	-1.641628
C	-1.523681	2.145778	0.128693
H	-2.425729	1.563800	-0.097866
H	-1.436346	2.246870	1.216393
C	-1.547345	3.495407	-0.562186
H	-1.613745	3.369519	-1.647363
H	-2.412706	4.075568	-0.222559
H	-0.636876	4.058133	-0.332979
C	2.623268	0.246215	0.125729
H	2.574057	1.318282	-0.102135
H	2.664671	0.120913	1.213628
C	3.802960	-0.412969	-0.562183
H	3.729474	-0.293701	-1.647651
H	4.739105	0.043540	-0.221548
H	3.830855	-1.482608	-0.331462
O	0.001439	-0.004954	1.868704

$Cu^{II}(OTf)_2$

Cu	0.000002	0.805440	0.000025
S	2.556462	0.693510	-0.000031
O	1.597773	0.799319	-1.189614
O	3.749222	1.513591	-0.000039
O	1.597821	0.799274	1.189597
C	3.076352	-1.103216	-0.000077
F	3.793087	-1.349111	1.090181
F	1.975496	-1.863292	-0.000062
F	3.793029	-1.349069	-1.090382
S	-2.556460	0.693510	0.000042
O	-3.749219	1.513593	0.000039
O	-1.597797	0.799291	-1.189565
O	-1.597796	0.799297	1.189645
C	-3.076355	-1.103214	0.000042
F	-1.975499	-1.863293	0.000059
F	-3.793075	-1.349083	1.090315
F	-3.793046	-1.349089	-1.090248

$Cu^{II}(OTf)^{\oplus}$

Cu	2.069068	-0.380816	0.000003
S	-0.223633	0.768829	-0.000007
O	-0.685508	2.130799	-0.000008
O	0.720084	0.309381	-1.170015
O	0.720088	0.309386	1.170015
C	-1.665923	-0.492487	0.000002
F	-1.105115	-1.692778	0.000005
F	-2.362253	-0.281339	1.091226
F	-2.362256	-0.281348	-1.091223

Cu^{II}(OTf) (TMG)$^{\oplus}$

Cu	0.031913	-0.115765	-0.385862
S	2.352267	0.670021	0.347267
O	2.997512	1.815700	0.944244
O	1.265719	-0.061556	1.160098
O	1.605760	0.867776	-0.987706
C	3.630508	-0.656065	-0.025264
F	2.990213	-1.694259	-0.570820
F	4.514567	-0.162936	-0.874689
F	4.205381	-1.015425	1.109491
N	-1.680147	-0.778930	-0.589190
H	-1.801627	-1.745702	-0.880876
C	-2.836627	-0.209203	-0.175118
N	-2.924388	1.137735	-0.092311
N	-3.896987	-0.975059	0.163308
C	-3.720615	1.811731	0.942702
H	-4.042474	1.095715	1.698639
H	-3.086827	2.562255	1.425875
H	-4.594048	2.314961	0.515497
C	-2.091599	2.028568	-0.907882
H	-1.218289	2.389792	-0.348540
H	-1.766145	1.520909	-1.817170
H	-2.691440	2.897709	-1.193281
C	-3.725753	-2.370577	0.585652
H	-2.751310	-2.508825	1.058192
H	-4.498249	-2.604817	1.322837
H	-3.834940	-3.062517	-0.259051
C	-5.286476	-0.549146	-0.054410
H	-5.812164	-1.353207	-0.578946
H	-5.799753	-0.353771	0.892561
H	-5.313837	0.344474	-0.677667

Cu^{II}(OTf) (TMG)$_2$$^{\oplus}$

Cu	-0.091080	-0.528986	0.004314
S	0.136903	1.934523	-0.896137
O	0.086070	2.641492	-2.161197
O	-1.125445	1.225766	-0.427035
O	1.219291	0.875729	-0.713399
C	0.518721	3.177382	0.447884
F	0.315994	2.600600	1.642768
F	1.797285	3.536586	0.346078
F	-0.272689	4.231212	0.321738
N	1.247258	-1.901809	0.313084
H	1.002988	-2.823469	-0.037682
C	2.554311	-1.635601	0.262412
N	3.061156	-0.636452	1.030672
N	3.407649	-2.342052	-0.525979
C	4.065168	0.302091	0.520042
H	4.359643	0.027439	-0.492146
H	3.620657	1.302698	0.481712
H	4.948928	0.321661	1.166556
C	2.340372	-0.173062	2.212584
H	1.686287	0.674172	1.976923
H	1.743076	-0.986599	2.625186
H	3.070665	0.153571	2.959724
C	2.917454	-3.075854	-1.690383
H	2.031341	-2.583556	-2.097482
H	3.695331	-3.073410	-2.459485
H	2.678572	-4.120469	-1.447991
C	4.798585	-2.606570	-0.149665
H	4.976632	-3.687564	-0.176322
H	5.496864	-2.120671	-0.840297
H	4.985254	-2.247397	0.862281
N	-1.642472	-1.590687	0.449079
H	-1.559372	-2.185543	1.267833
C	-2.894515	-1.239497	0.155975
N	-3.195909	-0.788574	-1.089525
N	-3.899637	-1.329891	1.069785
C	-4.118237	0.329991	-1.306599
H	-4.528098	0.671876	-0.356796
H	-3.561509	1.159544	-1.755788
H	-4.936925	0.040549	-1.974056
C	-2.339044	-1.098211	-2.230177
H	-1.629064	-0.284773	-2.426105

H	-1.792488	-2.023280	-2.042476
H	-2.968418	-1.226574	-3.116668
C	-3.607970	-1.350018	2.501067
H	-2.705000	-0.770839	2.707331
H	-4.443215	-0.887968	3.035295
H	-3.482676	-2.373074	2.882458
C	-5.271746	-1.686664	0.699119
H	-5.577236	-2.573075	1.266863
H	-5.968633	-0.871360	0.922826
H	-5.322269	-1.920805	-0.364195

$Cu^{II}(OTf)_2(TMG)$

Cu	0.053441	-0.326057	-0.468907
S	-2.904647	-1.038800	-0.941229
O	-3.260291	0.394085	-0.976748
O	-3.780338	-2.004176	-1.589120
O	-1.426465	-1.284360	-1.284794
C	-2.912008	-1.450223	0.877884
F	-2.540025	-2.708958	1.089531
F	-2.027205	-0.633446	1.517847
F	-4.119284	-1.241101	1.400344
S	2.112697	-1.280762	0.838479
O	2.557873	-1.766770	2.131416
O	1.043204	-2.048538	0.091928
O	1.647813	0.169273	0.747496
C	3.586875	-1.312290	-0.304622
F	3.191901	-0.952825	-1.536962
F	4.496986	-0.436586	0.128226
F	4.111706	-2.531116	-0.344564
N	-0.565097	1.445047	-0.874721
H	-1.576160	1.423562	-1.023461
C	-0.114839	2.536335	-0.272322
N	1.205265	2.865736	-0.351699
N	-0.943515	3.370727	0.418979
C	1.945514	3.369350	0.803524
H	1.272271	3.510649	1.649104
H	2.701321	2.629121	1.090088
H	2.440356	4.320069	0.571790
C	2.033446	2.347951	-1.436552
H	2.574839	1.447350	-1.132112
H	1.404861	2.110424	-2.295206
H	2.759191	3.119349	-1.718272
C	-2.237272	2.889471	0.905326
H	-2.182654	1.825952	1.138150
H	-2.489809	3.441106	1.816201
H	-3.035127	3.039814	0.167154
C	-0.759439	4.819778	0.438178
H	-1.661529	5.305080	0.045659
H	-0.584016	5.186373	1.456876
H	0.086532	5.097128	-0.191338

$Cu^{II}(OTf)_2(TMG)_2$

Cu	0.411996	-0.218049	0.177635
S	2.816215	-2.124010	0.758169
O	3.948875	-2.369809	1.647661
O	2.063615	-3.265508	0.220402
O	1.925584	-0.993618	1.280397
C	3.542212	-1.320437	-0.761274
F	2.543042	-0.994228	-1.630782
F	4.190992	-0.188792	-0.457244
F	4.376919	-2.148346	-1.387359
S	-2.020785	1.519676	-1.132055
O	-1.414549	2.831839	-0.864574
O	-2.985314	1.397396	-2.226639
O	-1.030317	0.369353	-1.133575
C	-3.003693	1.159436	0.410461
F	-3.632286	-0.024350	0.311673
F	-2.188220	1.100382	1.494037
F	-3.910907	2.111563	0.629286
N	-0.666960	-1.728766	0.722435
H	-0.486581	-2.038400	1.672695
C	-1.665793	-2.346265	0.124125
N	-1.702162	-2.477935	-1.230010
N	-2.701209	-2.887696	0.840377
C	-2.917108	-2.184389	-1.990457
H	-3.769560	-2.087013	-1.319141
H	-2.793174	-1.230609	-2.516915
H	-3.117172	-2.980999	-2.716623
C	-0.465873	-2.458045	-2.009164
H	-0.280873	-1.463248	-2.428849
H	0.373838	-2.756050	-1.379840
H	-0.567797	-3.173786	-2.833435
C	-2.979862	-2.427359	2.193130
H	-2.692352	-1.379751	2.291804

H	-4.055802	-2.511118	2.380425
H	-2.452914	-3.024413	2.952109
C	-3.382288	-4.109532	0.428616
H	-3.292209	-4.867597	1.217520
H	-4.448470	-3.928049	0.242022
H	-2.923629	-4.498073	-0.480895
N	1.451426	1.392841	-0.181807
H	2.036866	1.342331	-1.008926
C	1.258478	2.614945	0.263107
N	0.798009	2.826079	1.533619
N	1.530134	3.714810	-0.500535
C	-0.220012	3.838297	1.812462
H	-0.451566	4.396422	0.907613
H	-1.142095	3.342284	2.136370
H	0.114492	4.521585	2.602788
C	0.932021	1.785555	2.545816
H	0.034574	1.151703	2.597218
H	1.793202	1.153718	2.329289
H	1.064090	2.261755	3.523966
C	1.492501	3.624457	-1.953474
H	0.770418	2.862842	-2.249897
H	1.154523	4.584579	-2.356580
H	2.480483	3.399052	-2.382425
C	2.060973	4.950594	0.058496
H	3.008085	5.202076	-0.436460
H	1.364322	5.786955	-0.083445
H	2.251532	4.824417	1.124869

Cu^{II}(OTf)$_2$(THF)

Cu	0.182169	0.249767	-0.209221
S	-2.552559	-0.858593	-0.803044
O	-3.177724	0.284852	-0.121319
O	-3.314757	-1.634117	-1.770257
O	-1.148971	-0.505627	-1.355139
C	-2.090943	-2.010817	0.590445
F	-1.435597	-3.075468	0.143250
F	-1.273323	-1.332889	1.448750
F	-3.174315	-2.389436	1.260741
S	2.491809	-0.577871	0.610225
O	3.148167	-1.356757	1.641452
O	1.519006	-1.277515	-0.328760
O	1.713868	0.668200	1.035499
C	3.807061	0.104464	-0.526310
F	3.212410	0.841119	-1.474488
F	4.640713	0.869211	0.172989
F	4.469803	-0.899400	-1.090240
C	-1.415942	2.687445	-0.975947
O	-0.614629	2.012096	0.059036
C	-1.182010	2.261799	1.390036
C	-1.964443	3.553578	1.202842
C	-2.543203	3.384409	-0.214487
H	-0.732904	3.381952	-1.473182
H	-1.761650	1.927164	-1.674464
H	-1.833387	1.420567	1.639748
H	-0.335682	2.316449	2.076153
H	-2.740252	3.671486	1.964198
H	-1.291354	4.416786	1.253803
H	-3.421463	2.735725	-0.189552
H	-2.814219	4.337492	-0.676794

Cu^{II}(OTf)$_2$(THF)$_2$

Cu	-0.002086	0.004531	-0.006828
S	2.923710	0.444389	-0.725626
O	3.943422	0.098269	-1.707585
O	2.951773	1.758402	-0.069207
O	1.515296	0.071176	-1.228173
C	3.131212	-0.760316	0.687222
F	2.129584	-0.560780	1.593495
F	3.057605	-2.026402	0.268957
F	4.291348	-0.562676	1.304625
S	-2.918668	-0.439480	0.753795
O	-2.947340	-1.765344	0.121702
O	-3.928658	-0.080434	1.741014
O	-1.506366	-0.049487	1.231321
C	-3.145627	0.739830	-0.677134
F	-3.067943	2.013039	-0.282651
F	-2.152764	0.525732	-1.591028
F	-4.311622	0.530533	-1.279003
C	-0.208687	-2.578131	-1.408795
O	0.117813	-1.955552	-0.127578
C	-0.132494	-2.875873	0.984759
C	-0.730607	-4.120194	0.330914
C	-0.161688	-4.068635	-1.098852
H	-1.212298	-2.250705	-1.695401

H	0.535831	-2.220325	-2.120903
H	0.840258	-3.055169	1.450476
H	-0.802005	-2.377068	1.683148
H	-0.455941	-5.033208	0.866518
H	-1.819777	-4.031533	0.308021
H	0.873861	-4.426378	-1.121484
H	-0.749216	-4.653101	-1.812132
C	0.291249	2.585652	1.388285
O	-0.112018	1.964778	0.121530
C	0.095051	2.876703	-0.998145
C	0.075883	4.247474	-0.335185
C	0.801465	3.972105	0.994509
H	-0.601356	2.599349	2.017838
H	1.062923	1.958622	1.838628
H	1.057431	2.647546	-1.456693
H	-0.719510	2.689873	-1.698854
H	0.578871	5.003372	-0.944352
H	-0.956948	4.568120	-0.158256
H	1.881057	3.929750	0.829203
H	0.584857	4.719743	1.762335

Cu^{II}(OTf)$_2$(PPh$_3$)

Cu	0.525176	0.192622	0.854569
S	1.165689	-2.585554	0.131447
O	0.750271	-3.964268	0.362635
O	0.868111	-1.937735	-1.160617
O	0.780948	-1.660717	1.310390
C	3.026111	-2.563946	0.258144
F	3.477301	-1.325051	0.013320
F	3.412821	-2.938232	1.477814
F	3.542080	-3.399410	-0.648239
S	2.139519	2.252024	1.027604
O	2.526471	3.441644	1.765582
O	2.371889	0.895398	1.642448
O	0.695700	2.190865	0.517689
C	3.125966	2.216006	-0.557466
F	2.711761	1.181843	-1.302556
F	2.931538	3.349415	-1.230329
F	4.418149	2.071318	-0.276711
P	-1.610426	0.107169	-0.016097
C	-2.794666	0.992231	1.054738
C	-1.597366	0.960250	-1.625469
C	-2.264774	-1.568462	-0.261116
C	-4.092220	0.503873	1.272785
C	-2.391559	2.199950	1.651503
C	-2.580573	1.898292	-1.971361
C	-0.554681	0.667179	-2.521699
C	-2.791995	-1.979329	-1.493534
C	-2.219018	-2.465591	0.818219
C	-4.978040	1.220822	2.078251
H	-4.405782	-0.431070	0.819394
C	-3.284761	2.909735	2.453067
H	-1.388752	2.581310	1.483996
C	-2.523830	2.534969	-3.211857
H	-3.380853	2.133216	-1.276545
C	-0.509365	1.307358	-3.758935
H	0.203256	-0.061904	-2.252640
C	-3.274690	-3.279591	-1.639638
H	-2.814402	-1.293529	-2.334252
C	-2.701726	-3.762498	0.662465
H	-1.771869	-2.164731	1.760130
C	-4.576517	2.421470	2.668094
H	-5.981457	0.839291	2.244913
H	-2.968751	3.841593	2.913083
C	-1.490362	2.241377	-4.104178
H	-3.285675	3.262045	-3.478532
H	0.296765	1.079815	-4.450443
C	-3.229576	-4.170116	-0.564948
H	-3.677486	-3.598463	-2.596747
H	-2.641535	-4.460199	1.491847
H	-5.268247	2.974784	3.297189
H	-1.447095	2.742793	-5.067123
H	-3.593301	-5.186400	-0.687258

Cu^{II}(OTf)$_2$(PPh$_3$)$_2$

Cu	0.015709	0.026505	-0.275732
S	0.453948	3.119919	-0.163914
O	0.540913	4.098016	0.918019
O	1.655927	2.780124	-0.938740
O	-0.339739	1.872321	0.263467
C	-0.755967	3.827802	-1.392154
F	-0.963158	2.951868	-2.391148
F	-1.927336	4.074026	-0.793416
F	-0.278588	4.962807	-1.908942

S	-0.621393	-1.895329	-2.592923
O	-0.343688	-3.229534	-3.115424
O	-2.010384	-1.454783	-2.404760
O	0.272815	-1.575990	-1.380473
C	0.082709	-0.683947	-3.821290
F	0.030359	0.560525	-3.289208
F	1.359439	-0.967651	-4.096360
F	-0.624528	-0.690661	-4.951534
P	-2.086209	-0.457010	0.653498
C	-1.850733	0.014306	2.414066
C	-3.459164	0.566801	0.043792
C	-2.571764	-2.212095	0.647040
C	-1.416500	-0.927821	3.359994
C	-1.930884	1.370043	2.780160
C	-4.595399	0.826379	0.827752
C	-3.343743	1.143080	-1.228684
C	-3.906257	-2.626826	0.549197
C	-1.547698	-3.169145	0.728522
C	-1.075975	-0.521327	4.651036
H	-1.335587	-1.975829	3.090911
C	-1.596631	1.767447	4.074120
H	-2.227729	2.112231	2.048384
C	-5.609960	1.645538	0.333846
H	-4.676386	0.404607	1.825772
C	-4.356797	1.970272	-1.712237
H	-2.467408	0.946655	-1.833545
C	-4.211396	-3.988142	0.547080
H	-4.700894	-1.893103	0.458330
C	-1.860606	-4.527365	0.735657
H	-0.509280	-2.858479	0.760966
C	-1.164893	0.824814	5.011811
H	-0.741731	-1.260147	5.374565
H	-1.660626	2.818144	4.342505
C	-5.489657	2.219003	-0.935516
H	-6.488289	1.843410	0.942031
H	-4.253128	2.425489	-2.692732
C	-3.192085	-4.938581	0.643566
H	-5.246876	-4.306226	0.462613
H	-1.058831	-5.257377	0.794438
H	-0.896812	1.138688	6.016920
H	-6.276432	2.866030	-1.313817
H	-3.435039	-5.997620	0.634258
P	2.226435	-0.251404	0.464911
C	2.140844	-1.900200	1.273348
C	3.519333	-0.380084	-0.807018
C	2.769592	0.925280	1.745466
C	1.874048	-2.019958	2.646274
C	2.169322	-3.057459	0.473970
C	4.684154	-1.138533	-0.602443
C	3.329781	0.295658	-2.020817
C	4.105941	1.327619	1.881275
C	1.794205	1.442961	2.612330
C	1.646213	-3.276365	3.210104
H	1.834827	-1.136019	3.274383
C	1.950855	-4.309970	1.046455
H	2.336166	-2.975144	-0.593933
C	5.652025	-1.211821	-1.603237
H	4.824485	-1.679821	0.328981
C	4.300660	0.213365	-3.019267
H	2.435121	0.886631	-2.176469
C	4.459744	2.229912	2.884050
H	4.862315	0.949592	1.200651
C	2.156671	2.338150	3.616997
H	0.751681	1.173444	2.486789
C	1.684479	-4.423133	2.414204
H	1.440622	-3.356382	4.274226
H	1.976234	-5.195751	0.417948
C	5.459664	-0.536585	-2.812312
H	6.551526	-1.799654	-1.442414
H	4.144361	0.730964	-3.961130
C	3.488373	2.733033	3.753732
H	5.494729	2.545982	2.981346
H	1.389537	2.739381	4.271905
H	1.505855	-5.399600	2.856052
H	6.211731	-0.601150	-3.593986
H	3.768581	3.443040	4.527059

$Cu^{III}(OTf)_3(TMG)$

C	3.599038	0.976184	-1.127581
F	3.664534	1.580775	-2.313507
F	3.514965	-0.337909	-1.298341
F	4.682324	1.272752	-0.409028
O	0.961294	1.020067	-1.104492
O	2.098121	1.007303	1.128087

O	2.109419	3.068719	-0.336654
S	2.096963	1.608724	-0.206385
Cu	-0.332962	-0.172571	-0.452158
C	2.761972	-2.715651	0.703226
F	3.240444	-1.554145	1.150113
F	3.339022	-3.017029	-0.460803
F	3.030353	-3.672207	1.595534
O	0.827990	-1.637101	-0.726973
O	0.486076	-3.972625	0.131844
O	0.374058	-1.967522	1.692814
S	0.905183	-2.624332	0.488588
C	-4.327426	-0.866941	0.181609
F	-5.522806	-0.732813	-0.394410
F	-4.017216	0.279794	0.807832
F	-4.362300	-1.852686	1.073100
O	-3.004009	-0.065450	-2.013452
O	-1.782596	-1.367642	-0.245960
O	-3.426568	-2.543743	-1.687308
S	-3.060017	-1.247884	-1.137827
N	-1.464580	1.311502	-0.130052
H	-1.968639	1.498586	-0.996621
C	-1.069756	2.378319	0.574614
N	-0.647735	2.199834	1.839862
N	-1.178067	3.625986	0.046500
C	0.306572	3.104326	2.486201
H	0.753186	3.768308	1.747211
H	1.108338	2.495651	2.909759
H	-0.176225	3.683343	3.281589
C	-0.908567	0.939165	2.548684
H	-0.060066	0.255962	2.445069
H	-1.801034	0.464708	2.141482
H	-1.061883	1.169800	3.606943
C	-0.905958	3.874059	-1.370294
H	-0.421836	3.005042	-1.813987
H	-0.211465	4.716943	-1.448280
H	-1.829235	4.117020	-1.910264
C	-1.596684	4.779707	0.835838
H	-2.381163	5.313548	0.287782
H	-0.763325	5.470266	1.012710
H	-2.001451	4.450142	1.793559

Cu^{I}(OTf)(TMG)$_3$

C	-2.060840	-0.130264	-2.211709
F	-1.684616	1.169246	-2.157380
F	-2.980882	-0.320398	-1.243452
F	-2.661023	-0.333298	-3.389279
O	0.445553	-0.390922	-1.377316
O	-1.094759	-2.324018	-1.059155
O	-0.259732	-1.730507	-3.353523
S	-0.581094	-1.290863	-1.987688
Cu	0.330707	0.246801	1.013301
N	0.842497	-3.801011	1.202080
N	2.372369	-2.375806	0.181891
N	0.860826	-1.499559	1.743602
C	1.330984	-2.521910	1.060391
C	-0.535975	-4.022000	1.604225
C	1.696152	-4.976822	1.137997
C	3.207330	-1.189859	0.257230
C	2.321126	-3.003658	-1.140589
H	0.290705	-1.821926	2.521663
H	-0.879137	-4.969465	1.175307
H	-0.656784	-4.081472	2.698343
H	-1.159748	-3.229099	1.194293
H	1.598885	-5.561317	2.063852
H	1.429080	-5.626103	0.293091
H	2.737302	-4.670649	1.029394
H	4.200302	-1.432865	-0.139799
H	2.776856	-0.366793	-0.319698
H	3.298256	-0.872494	1.296783
H	2.245913	-2.228325	-1.909673
H	3.217295	-3.611350	-1.321016
H	1.430686	-3.625769	-1.223540
N	-3.571181	2.336766	0.705057
N	-3.327335	0.239130	1.711058
N	-1.416306	1.354095	0.955502
C	-2.704797	1.312212	1.112238
C	-2.968686	3.602908	0.314821
C	-4.708642	1.965698	-0.145209
C	-2.597193	-1.008168	1.853896
C	-4.385880	0.448808	2.691509
H	-1.143512	2.111649	0.336213
H	-3.754379	4.363464	0.264427
H	-2.232692	3.916055	1.060507
H	-2.480344	3.552369	-0.673367

H	-4.416794	1.949509	-1.205551
H	-5.066706	0.971785	0.118711
H	-5.520192	2.689097	-0.009827
H	-3.321487	-1.814561	2.015855
H	-2.036825	-1.217253	0.942877
H	-1.899908	-0.984266	2.705275
H	-4.000226	0.307938	3.713706
H	-4.779272	1.461756	2.607622
H	-5.202584	-0.266438	2.533494
N	1.294247	1.899646	0.006315
H	0.933265	1.816538	-0.941419
C	2.541879	2.257055	0.026714
N	3.150036	2.563954	1.238571
N	3.362542	2.344313	-1.094109
C	4.457101	1.988936	1.553901
H	4.989126	1.727382	0.639447
H	4.344682	1.075173	2.157506
H	5.059091	2.705602	2.124996
C	2.279048	2.684722	2.401337
H	1.939851	1.702178	2.765019
H	1.393197	3.266069	2.142906
H	2.833092	3.193019	3.198153
C	2.869208	1.808421	-2.358715
H	2.319446	0.877789	-2.202616
H	3.726769	1.605878	-3.008709
H	2.208793	2.520792	-2.880958
C	4.264043	3.484388	-1.252676
H	3.824565	4.235220	-1.928152
H	5.220218	3.159300	-1.680123
H	4.447403	3.958283	-0.288697

Cu^{III}-dimer

Cu	-1.799328	0.080268	-0.105132
O	-0.600654	0.410750	-1.550145
O	0.481080	-0.310024	-1.579913
S	-1.959946	3.211188	-0.627043
O	-2.014765	1.950403	0.256201
O	-1.954314	4.402591	0.222670
O	-0.965364	3.140576	-1.712489
C	-3.608457	3.133450	-1.499961
F	-3.590671	3.979052	-2.533474
F	-3.791650	1.884160	-1.963610
F	-4.593227	3.458079	-0.676357
N	-1.187382	-1.697252	-0.150613
H	-0.793470	-1.935909	-1.061454
C	-1.577178	-2.759292	0.561758
N	-1.659723	-2.684368	1.907785
N	-1.801990	-3.948491	-0.046983
C	-2.732463	-3.340498	2.653352
H	-3.381375	-3.885761	1.969373
H	-3.337062	-2.571278	3.146760
H	-2.324460	-4.029448	3.401549
C	-0.865711	-1.712214	2.652651
H	-1.448671	-0.811219	2.867179
H	0.024037	-1.461412	2.074597
H	-0.555324	-2.175314	3.595215
C	-2.183977	-4.019030	-1.457068
H	-2.724341	-3.115906	-1.741356
H	-2.850075	-4.877097	-1.589680
H	-1.298372	-4.143489	-2.089685
C	-1.403401	-5.213814	0.569436
H	-0.761120	-5.754414	-0.132065
H	-2.280400	-5.826308	0.810456
H	-0.824617	-5.018359	1.470988
Cu	1.782329	0.046197	-0.228038
S	4.364662	1.340560	0.140469
O	3.325618	0.457645	0.873820
O	4.067801	1.416173	-1.300380
O	4.638338	2.583263	0.868273
C	5.882074	0.270385	0.318240
F	6.924487	0.902188	-0.224840
F	6.122385	0.041954	1.610535
F	5.688019	-0.889798	-0.310283
N	1.097613	1.790783	-0.164089
H	0.766591	2.110062	-1.075472
C	1.396797	2.784935	0.681669
N	1.452489	2.540738	2.008944
N	1.543564	4.048907	0.223411
C	2.377450	3.252783	2.888892
H	3.037791	3.885104	2.298687
H	3.000842	2.514627	3.403538
H	1.835393	3.851896	3.629561
C	0.812792	1.358303	2.574268
H	1.511019	0.513845	2.613732

H	-0.058792	1.097503	1.973511
H	0.479574	1.596367	3.589013
C	2.013268	4.307850	-1.140076
H	2.638709	3.481179	-1.481539
H	2.621025	5.217307	-1.120763
H	1.162431	4.450008	-1.815280
C	1.021519	5.203993	0.953704
H	0.360777	5.763703	0.285918
H	1.838111	5.850321	1.296178
H	0.427839	4.869399	1.803225
S	1.823371	-3.139094	-0.494023
O	1.940103	-1.808249	0.264774
O	1.649996	-4.240676	0.455343
O	0.925841	-3.091994	-1.660872
C	3.537173	-3.264221	-1.210042
F	3.617571	-4.331971	-2.005894
F	3.785286	-2.158052	-1.927077
F	4.436845	-3.366646	-0.232810
S	-4.648145	-0.253186	1.131174
O	-3.111891	-0.359481	1.242781
O	-5.243766	-0.887015	2.308070
O	-5.129846	1.035575	0.651128
C	-4.962802	-1.436254	-0.279248
F	-6.248117	-1.441339	-0.613615
F	-4.233701	-1.075762	-1.363182
F	-4.591904	-2.686437	0.058582

Cu^{II}-dimer

Cu	1.781191	0.172894	-0.568949
O	0.621541	-0.326035	-2.022363
O	-0.620441	0.324948	-2.022394
S	3.469706	-2.490736	-0.966377
O	2.307975	-1.837453	-0.248604
O	3.270651	-3.938860	-1.134693
O	3.984444	-1.716334	-2.104807
C	4.856266	-2.378518	0.318128
F	5.621512	-3.469743	0.289411
F	5.644299	-1.305565	0.098339
F	4.351928	-2.246805	1.569629
N	3.468058	0.625684	0.440508
H	3.724646	-0.059713	1.142206
C	4.351565	1.568671	0.281267
N	4.354620	2.357790	-0.843473
N	5.334231	1.852921	1.222922
C	4.441988	3.809347	-0.733373
H	4.796675	4.090007	0.258997
H	3.451338	4.261824	-0.891595
H	5.130706	4.213087	-1.485511
C	3.793010	1.864020	-2.099275
H	2.731392	2.119156	-2.208610
H	3.910897	0.779772	-2.166404
H	4.347796	2.325019	-2.924937
C	5.253542	1.187112	2.518174
H	4.242768	1.273650	2.924664
H	5.943466	1.681866	3.207976
H	5.525866	0.122088	2.458528
C	6.711990	2.069029	0.768560
H	7.279528	1.127368	0.799774
H	7.207887	2.801222	1.415003
H	6.715421	2.437205	-0.256177
N	0.724800	1.828509	-0.330697
H	0.062466	1.811786	-1.115126
C	0.738684	2.924293	0.370889
N	1.174258	2.912901	1.681407
N	0.330507	4.141939	-0.124369
C	2.077996	3.926531	2.199132
H	2.144955	4.753947	1.491630
H	3.087383	3.510649	2.341998
H	1.724994	4.315067	3.163489
C	1.064961	1.689689	2.455209
H	1.962405	1.062929	2.381415
H	0.215749	1.112892	2.085528
H	0.894816	1.948579	3.507932
C	0.110677	4.270030	-1.565282
H	0.892199	3.735029	-2.109626
H	0.160798	5.331981	-1.827279
H	-0.872564	3.886560	-1.859377
C	-0.533560	5.016329	0.678504
H	-1.567708	4.933407	0.323795
H	-0.202422	6.058054	0.589858
H	-0.492577	4.717800	1.726145
Cu	-1.779641	-0.173054	-0.568267
S	-3.469414	2.490052	-0.967232
O	-2.307144	1.837456	-0.249753

O	-3.271039	3.938223	-1.135916
O	-3.984223	1.715218	-2.105329
C	-4.855626	2.377661	0.317643
F	-5.621458	3.468452	0.288588
F	-5.643182	1.304200	0.098522
F	-4.350962	2.246823	1.569127
N	-3.466252	-0.625548	0.441865
H	-3.722234	0.059971	1.143672
C	-4.351161	-1.567106	0.281970
N	-4.354981	-2.355950	-0.842886
N	-5.334772	-1.850113	1.223150
C	-4.446330	-3.807280	-0.733464
H	-4.801205	-4.087410	0.258985
H	-3.456999	-4.262330	-0.892483
H	-5.136554	-4.208825	-1.485408
C	-3.792236	-1.862979	-2.098480
H	-2.730866	-2.119331	-2.207344
H	-3.908923	-0.778617	-2.165844
H	-4.347198	-2.323437	-2.924318
C	-5.253122	-1.185241	2.518837
H	-4.242646	-1.274078	2.925557
H	-5.944243	-1.679041	3.208131
H	-5.523302	-0.119638	2.459805
C	-6.712957	-2.061400	0.767677
H	-7.276964	-1.117570	0.797512
H	-7.212108	-2.791145	1.414365
H	-6.716898	-2.430561	-0.256703
N	-0.723570	-1.828647	-0.329731
H	-0.060729	-1.811892	-1.113742
C	-0.739208	-2.925193	0.370662
N	-1.176133	-2.914747	1.680653
N	-0.331770	-4.142725	-0.125546
C	-2.079756	-3.928943	2.197327
H	-2.147869	-4.754920	1.488254
H	-3.088747	-3.512761	2.342028
H	-1.726064	-4.319611	3.160590
C	-1.065554	-1.692952	2.456446
H	-1.962694	-1.065546	2.384369
H	-0.216279	-1.116042	2.087116
H	-0.894824	-1.953658	3.508642
C	-0.111172	-4.269606	-1.566470
H	-0.891758	-3.733210	-2.110764
H	-0.162280	-5.331247	-1.829545
H	0.872663	-3.886898	-1.859582
C	0.531389	-5.018356	0.676954
H	1.565829	-4.935457	0.323067
H	0.199899	-6.059850	0.586943
H	0.489838	-4.721009	1.724915

INT1

C	-1.089405	-2.888463	-0.686097
O	-1.668069	-4.081744	-0.812165
O	-1.544314	-2.057531	0.123153
C	-2.833760	-4.347694	-0.010377
H	-3.106010	-5.378853	-0.231153
H	-3.640934	-3.667233	-0.292055
H	-2.605014	-4.226237	1.051823
C	0.054340	-2.760602	-1.587548
H	0.208171	-3.617781	-2.233420
C	0.856512	-1.681495	-1.644015
H	0.678870	-0.839295	-0.982268
C	2.037894	-1.497755	-2.561496
C	2.203446	-2.593686	-3.618657
H	3.082018	-2.388139	-4.236500
H	1.326492	-2.628575	-4.273082
H	2.330915	-3.588049	-3.177452
C	3.279584	-1.262140	-1.687143
C	4.186674	-2.287863	-1.391243
C	3.509889	0.015116	-1.154123
C	5.307177	-2.039875	-0.592915
H	4.038548	-3.284311	-1.795406
C	4.623004	0.261391	-0.349330
H	2.826891	0.821746	-1.394366
C	5.527458	-0.765914	-0.063979
H	6.012068	-2.842413	-0.392926
H	4.800025	1.266820	0.024118
H	6.407081	-0.570449	0.543330
Cu	-1.104458	-0.110908	0.772326
S	0.040398	2.102260	-1.276978
O	-1.311485	2.668229	-1.439399
O	0.186432	1.258875	-0.008448
O	0.668859	1.474076	-2.443392
C	1.124306	3.543510	-0.814446
F	2.385229	3.121889	-0.619747

F	1.107569	4.455905	-1.777682
F	0.680285	4.091347	0.328895
N	0.167972	-0.812219	2.027973
H	0.207831	-1.826214	2.054139
C	1.306344	-0.240306	2.428416
N	1.327306	1.075830	2.754636
N	2.456675	-0.957012	2.533044
C	2.472446	1.932421	2.434378
H	3.185237	1.386385	1.817424
H	2.114558	2.789037	1.860227
H	2.968649	2.295605	3.341533
C	0.100343	1.794560	3.089043
H	-0.300312	2.311745	2.210289
H	-0.643995	1.096711	3.475170
H	0.329729	2.534652	3.862600
C	2.630946	-2.205748	1.796415
H	2.076278	-2.173182	0.858198
H	3.687374	-2.314665	1.545466
H	2.314931	-3.076423	2.389010
C	3.486267	-0.673885	3.532894
H	3.703536	-1.593885	4.087388
H	4.410975	-0.325033	3.059926
H	3.131323	0.076926	4.238537
N	-2.709435	0.743526	0.173511
H	-2.510935	1.569171	-0.392886
C	-3.962431	0.328137	0.101240
N	-4.457681	-0.546346	1.024551
N	-4.805072	0.765373	-0.881828
C	-5.490994	-1.527200	0.701790
H	-5.690691	-1.524189	-0.370298
H	-5.140928	-2.525003	0.989949
H	-6.424385	-1.326101	1.240737
C	-3.783641	-0.747200	2.301665
H	-3.053231	-1.566048	2.249606
H	-3.273033	0.170202	2.601085
H	-4.531843	-0.994332	3.061503
C	-4.253224	1.337973	-2.112421
H	-3.329711	0.825810	-2.386745
H	-4.986280	1.200492	-2.912351
H	-4.037975	2.409188	-2.011012
C	-6.197495	1.129947	-0.602242
H	-6.340333	2.196557	-0.813138
H	-6.890276	0.555938	-1.227668
H	-6.433633	0.956722	0.447797
H	1.839353	-0.548080	-3.076191

INT2

C	-1.223004	3.135736	-0.913117
O	-2.569459	3.386034	-0.859228
O	-0.406201	3.944728	-0.475331
C	-2.932096	4.618636	-0.225737
H	-4.020461	4.672508	-0.287822
H	-2.610083	4.626125	0.820803
H	-2.476566	5.470329	-0.738536
C	-0.936192	1.833157	-1.491579
H	-1.778201	1.323889	-1.946186
C	0.403041	1.517359	-1.884552
H	1.162065	2.159819	-1.449417
C	0.831535	0.495383	-2.693562
C	-0.102031	-0.428439	-3.428684
H	-0.102538	-1.409180	-2.936337
H	-1.126653	-0.046588	-3.456759
H	0.223001	-0.585941	-4.463780
C	2.282112	0.226208	-2.787587
C	2.913261	-0.035596	-4.017391
C	3.068412	0.208525	-1.618232
C	4.285940	-0.267607	-4.080891
H	2.330234	-0.029453	-4.934338
C	4.439022	-0.034340	-1.683425
H	2.579964	0.311844	-0.655251
C	5.056105	-0.265198	-2.914522
H	4.756578	-0.451678	-5.043394
H	5.020390	-0.069894	-0.765755
H	6.124285	-0.459117	-2.964962
Cu	-0.613165	0.552514	0.336659
S	1.007910	-2.436728	0.104037
O	-0.012329	-2.690196	-0.947116
O	0.976765	-1.030936	0.636777
O	2.349961	-2.978553	-0.112199
C	0.316863	-3.397486	1.544578
F	1.106916	-3.290316	2.629586
F	0.186970	-4.692986	1.242945
F	-0.903320	-2.918289	1.879734
N	0.435632	1.860258	1.359786

H	0.485827	2.771298	0.905002
C	1.441455	1.620125	2.167483
N	1.377792	0.626887	3.106838
N	2.611787	2.351382	2.132528
C	2.511848	-0.271876	3.328331
H	3.390089	0.099851	2.801119
H	2.271458	-1.262422	2.933684
H	2.739367	-0.350233	4.398867
C	0.084137	0.074016	3.484761
H	-0.206405	-0.761506	2.839578
H	-0.677365	0.853267	3.420543
H	0.148876	-0.284439	4.518787
C	2.921924	3.152342	0.954717
H	2.687087	2.591252	0.049228
H	3.995659	3.364013	0.953509
H	2.378914	4.108892	0.932907
C	3.288642	2.761149	3.359253
H	3.202286	3.848898	3.495672
H	4.353962	2.501475	3.325426
H	2.832412	2.268019	4.217597
N	-1.997331	-0.748424	-0.090122
H	-1.560953	-1.617993	-0.402839
C	-3.282996	-0.684453	-0.292850
N	-4.055665	0.293874	0.297193
N	-3.957531	-1.579213	-1.104370
C	-5.043743	1.033144	-0.489014
H	-5.278793	0.487288	-1.402884
H	-4.640490	2.016090	-0.763935
H	-5.965729	1.170929	0.089027
C	-3.534701	1.022961	1.445512
H	-2.923108	1.883315	1.146248
H	-2.925464	0.357256	2.058928
H	-4.382729	1.383161	2.039965
C	-3.180626	-2.452112	-1.979654
H	-2.376616	-1.889126	-2.455068
H	-3.845276	-2.841094	-2.757215
H	-2.732232	-3.300128	-1.442471
C	-5.242372	-2.137155	-0.685076
H	-5.111073	-3.164626	-0.314690
H	-5.943367	-2.161473	-1.527860
H	-5.670126	-1.534399	0.115765

INT2'

C	-0.780572	-3.442742	0.901716
O	-1.962074	-3.964315	0.504850
O	-0.337243	-3.569871	2.034655
C	-2.702432	-4.652319	1.523398
H	-3.615288	-4.998011	1.037642
H	-2.128365	-5.497409	1.914735
H	-2.939827	-3.974111	2.348738
C	-0.141269	-2.685734	-0.191812
H	-0.642700	-2.696166	-1.153645
C	1.186032	-2.235083	-0.010937
H	1.632859	-2.492507	0.947404
C	1.983230	-1.547455	-0.963580
C	3.254794	-0.955924	-0.557432
C	3.558328	-0.692555	0.803005
C	4.224737	-0.586512	-1.519332
C	4.762369	-0.102580	1.172827
H	2.828202	-0.922453	1.572055
C	5.431792	-0.004340	-1.142848
H	4.039356	-0.779335	-2.570596
C	5.710228	0.244061	0.204033
H	4.960531	0.093094	2.223431
H	6.160786	0.255640	-1.905744
H	6.650378	0.703846	0.495202
C	1.527125	-1.420823	-2.395703
H	1.969786	-2.215153	-3.014544
H	0.441694	-1.491970	-2.488638
H	1.823662	-0.460858	-2.826986
Cu	-0.194634	-0.711683	0.426532
S	-2.447736	0.129662	-1.505297
O	-3.813118	0.634288	-1.645481
O	-2.042548	-0.038857	-0.047532
O	-1.989148	-0.964980	-2.375975
C	-1.362504	1.565367	-2.008319
F	-0.052363	1.209214	-1.970862
F	-1.643375	1.971533	-3.246580
F	-1.513466	2.607977	-1.167938
N	0.607017	0.883057	1.246169
H	1.566787	1.099534	0.991141
C	-0.092246	1.945166	1.592947
N	-1.271650	1.809470	2.274188
N	0.316741	3.222521	1.306025

C	-2.465634	2.551055	1.858187
H	-2.194239	3.349596	1.170070
H	-3.146052	1.871406	1.332103
H	-2.972759	2.978191	2.731167
C	-1.562127	0.570115	2.989057
H	-2.125523	-0.131538	2.362136
H	-0.631541	0.098151	3.306508
H	-2.165680	0.812013	3.871641
C	1.278209	3.461377	0.238664
H	1.136507	2.731138	-0.558586
H	1.100831	4.458867	-0.176433
H	2.318729	3.418779	0.594693
C	0.058986	4.348282	2.195962
H	1.010465	4.795598	2.513631
H	-0.537046	5.123488	1.697668
H	-0.476527	4.004284	3.081123

INT3

C	-2.733971	1.097726	-0.305890
C	-1.973491	0.434539	-1.463903
C	-2.427665	-0.747438	-2.066809
C	-0.757164	0.976460	-1.911136
C	-1.671384	-1.391334	-3.049849
H	-3.382145	-1.174556	-1.774682
C	-0.000805	0.335401	-2.893215
H	-0.385833	1.889764	-1.465030
C	-0.446099	-0.861875	-3.454861
H	-2.042808	-2.311617	-3.493708
H	0.957265	0.748895	-3.188286
H	0.164246	-1.375494	-4.191223
O	-0.769421	1.470501	0.999471
O	-2.058829	0.845030	0.966182
Cu	0.698440	0.225416	0.683465
S	2.967696	-0.872528	-1.510382
O	3.080682	0.552585	-1.907283
O	1.962137	-1.116982	-0.410036
O	2.929507	-1.868151	-2.582874
C	4.569325	-1.162560	-0.603892
F	4.688764	-2.447942	-0.224430
F	5.614764	-0.849663	-1.374042
F	4.611214	-0.397308	0.506498
N	-0.310373	-1.301848	1.358110
H	-1.305999	-1.097961	1.321160
C	0.016061	-2.564849	1.474721
N	1.268373	-2.934102	1.887926
N	-0.869786	-3.585058	1.214484
C	1.994996	-4.005454	1.205965
H	1.330806	-4.529482	0.518985
H	2.816017	-3.575225	0.625094
H	2.401274	-4.721150	1.931658
C	2.118643	-1.975638	2.584092
H	2.738504	-1.400981	1.887607
H	1.497745	-1.286885	3.160056
H	2.774019	-2.527011	3.268644
C	-2.058890	-3.319179	0.418629
H	-1.828997	-2.587387	-0.357147
H	-2.375465	-4.249333	-0.064937
H	-2.899683	-2.950135	1.027489
C	-0.914893	-4.794052	2.029558
H	-1.872867	-4.849865	2.565337
H	-0.814160	-5.693257	1.408894
H	-0.107913	-4.777942	2.762322
N	1.964718	1.710671	0.484303
H	2.569923	1.569195	-0.324940
C	1.760594	2.966379	0.798840
N	1.236522	3.314989	2.019508
N	2.067906	3.999399	-0.056539
C	0.128300	4.267064	2.101359
H	0.063497	4.850404	1.182646
H	-0.810739	3.714287	2.225399
H	0.272286	4.948951	2.947966
C	1.260239	2.345341	3.110420
H	0.369071	1.706533	3.089650
H	2.151133	1.721086	3.023278
H	1.294469	2.893862	4.059029
C	2.322947	3.717838	-1.465354
H	1.682050	2.906621	-1.812268
H	2.101047	4.618629	-2.046727
H	3.366255	3.426766	-1.651232
C	2.605509	5.266956	0.427712
H	3.637353	5.396195	0.072415
H	2.008528	6.113293	0.065194
H	2.609570	5.275972	1.517730
C	-4.048976	0.400179	-0.048266

H	-3.973975	-0.552927	0.472565
C	-5.267985	0.819346	-0.398844
H	-5.448582	1.755920	-0.915459
C	-6.448981	-0.020670	-0.090817
O	-6.428098	-1.115973	0.439443
O	-7.589621	0.593044	-0.486560
C	-8.795622	-0.143364	-0.235006
H	-9.606345	0.480915	-0.612595
H	-8.775277	-1.105437	-0.755716
H	-8.917924	-0.327566	0.836526
C	-2.891517	2.609628	-0.500178
H	-3.494961	3.025463	0.312019
H	-1.909888	3.084269	-0.476268
H	-3.369491	2.833317	-1.459000

INT4

C	-6.489753	0.916508	0.619600
O	-7.184155	0.825170	-0.544571
O	-7.024553	0.937409	1.711992
C	-8.609223	0.758556	-0.394093
H	-9.010967	0.696012	-1.406474
H	-8.895577	-0.122254	0.188751
H	-8.986342	1.650649	0.114964
C	-5.036912	0.977781	0.353497
H	-4.713924	0.963165	-0.679238
C	-4.169679	1.044116	1.369706
H	-4.559755	1.053878	2.385880
C	-2.667646	1.122417	1.256748
Cu	0.214415	-0.167611	-0.362399
O	-2.376426	0.965754	-0.153965
O	-1.005834	1.295551	-0.403087
S	1.481177	-2.953972	0.832820
O	2.001935	-3.275040	2.163650
O	0.228528	-3.605886	0.397311
O	1.529642	-1.466408	0.525278
C	2.758618	-3.590603	-0.362816
F	2.432353	-3.233872	-1.622189
F	3.964356	-3.085357	-0.078370
F	2.823795	-4.925455	-0.306445
N	-0.751152	-1.538879	-1.379967
H	-0.631785	-2.460598	-0.959454
C	-1.816652	-1.400048	-2.129932
N	-1.937429	-0.343097	-2.997364
N	-2.845896	-2.312107	-2.120239
C	-3.184385	0.411016	-3.098283
H	-4.000917	-0.144358	-2.637979
H	-3.079166	1.364362	-2.566472
H	-3.431212	0.605196	-4.149444
C	-0.753816	0.407569	-3.401367
H	-0.569616	1.253081	-2.729333
H	0.116760	-0.250614	-3.389821
H	-0.910073	0.779585	-4.421093
C	-2.930335	-3.291967	-1.043184
H	-2.562328	-2.861733	-0.112331
H	-3.979315	-3.577952	-0.910992
H	-2.345726	-4.197121	-1.263269
C	-3.562919	-2.677510	-3.337721
H	-3.365628	-3.730034	-3.584699
H	-4.645457	-2.551110	-3.210989
H	-3.227675	-2.058843	-4.170187
P	2.019263	1.413486	-0.188767
C	3.631233	0.642313	-0.696164
C	1.939195	3.031001	-1.119679
C	2.243540	1.885622	1.599984
C	4.853188	1.006839	-0.117720
C	3.598235	-0.334186	-1.697098
C	3.094347	3.644611	-1.621787
C	0.690310	3.643573	-1.301283
C	1.933299	3.168615	2.063011
C	2.635135	0.887727	2.505003
C	6.035598	0.405761	-0.552895
H	4.879122	1.747166	0.676741
C	4.780853	-0.933967	-2.128501
H	2.645607	-0.655539	-2.107832
C	3.001811	4.862935	-2.296437
H	4.063750	3.173583	-1.494449
C	0.607365	4.866243	-1.971483
H	-0.205406	3.151917	-0.932798
C	2.014461	3.454265	3.428632
H	1.626384	3.941644	1.365157
C	2.728920	1.186082	3.864206
H	2.835021	-0.118831	2.151620
C	6.000460	-0.561520	-1.560459
H	6.982126	0.688152	-0.099983

H	4.742767	-1.710992	-2.886210
C	1.759504	5.477527	-2.469846
H	3.902008	5.331094	-2.685698
H	-0.362943	5.336445	-2.108504
C	2.414437	2.466240	4.329865
H	1.768692	4.451337	3.784079
H	3.035916	0.410836	4.561016
H	6.920948	-1.035701	-1.889857
H	1.690480	6.426758	-2.994589
H	2.478676	2.690640	5.391052
C	-2.234457	2.520543	1.733276
H	-2.485720	2.662326	2.788997
H	-1.160395	2.653762	1.599404
H	-2.753722	3.276454	1.137038
C	-2.024647	-0.028661	2.036366
C	-2.636925	-1.291419	2.009532
C	-0.803270	0.107822	2.707309
C	-2.044595	-2.389126	2.629304
H	-3.583393	-1.409456	1.489837
C	-0.206013	-0.996161	3.322760
H	-0.296418	1.064970	2.742971
C	-0.820884	-2.245462	3.286315
H	-2.526732	-3.361844	2.584339
H	0.759054	-0.881420	3.803109
H	-0.329595	-3.104619	3.729661

INT4'

C	-5.601405	-0.039798	-0.373700
O	-6.085392	0.090429	0.888297
O	-6.221788	-0.578222	-1.271002
C	-7.390655	-0.470127	1.095098
H	-7.635155	-0.270900	2.139253
H	-8.121993	-0.001181	0.430512
H	-7.379866	-1.546945	0.900728
C	-4.241226	0.530737	-0.472001
H	-3.809729	0.973895	0.416626
C	-3.560059	0.446568	-1.617518
H	-4.046753	-0.017394	-2.473841
C	-2.173405	0.996335	-1.852522
C	-1.213346	-0.064686	-2.404448
C	-1.555255	-1.422335	-2.448262
C	0.074428	0.313167	-2.818125
C	-0.654295	-2.370088	-2.939094
H	-2.517813	-1.752606	-2.073804
C	0.976319	-0.630433	-3.311702
H	0.392180	1.347163	-2.740516
C	0.607431	-1.975022	-3.386139
H	-0.938133	-3.418557	-2.955294
H	1.971463	-0.318722	-3.608995
H	1.312168	-2.709974	-3.764040
C	-2.310995	2.202078	-2.805431
H	-2.750066	1.886150	-3.756195
H	-2.958886	2.955361	-2.347639
H	-1.335714	2.652394	-3.005562
Cu	0.508371	0.113990	0.164313
O	-1.628766	1.586512	-0.657799
O	-1.340655	0.546727	0.301824
S	3.478445	-0.001892	-1.035445
O	4.047833	-0.994028	-1.950150
O	3.157648	1.335665	-1.575495
O	2.370373	-0.577653	-0.164166
C	4.766540	0.306639	0.271752
F	4.283051	1.162385	1.188018
F	5.086485	-0.839672	0.888434
F	5.868054	0.832563	-0.270601
N	1.168833	1.954191	0.493071
H	1.920796	2.199041	-0.148691
C	0.426104	2.960436	0.888182
N	-0.384839	2.839759	1.987018
N	0.434516	4.176835	0.254026
C	-1.773025	3.295794	1.938082
H	-1.933144	3.920357	1.060331
H	-2.431325	2.423528	1.852541
H	-2.026824	3.860307	2.843336
C	-0.148760	1.754343	2.930352
H	-0.686838	0.845199	2.634945
H	0.918707	1.535514	2.978384
H	-0.498793	2.070036	3.920038
C	0.946172	4.272615	-1.106103
H	0.707547	3.358725	-1.651829
H	0.460923	5.117204	-1.606115
H	2.034452	4.427643	-1.136573
C	0.308129	5.437154	0.976339
H	1.220182	6.035236	0.845748

H	-0.544012	6.022869	0.608579
H	0.170663	5.240778	2.039928
P	-0.040127	-2.165590	0.837871
O	-1.595185	-2.759598	0.741908
O	0.135784	-2.175875	2.527818
O	0.732251	-3.558915	0.348295
C	-2.651006	-2.087457	1.477179
C	1.400610	-1.723059	3.052096
C	1.869820	-3.549959	-0.557789
H	-2.493672	-2.257419	2.548034
H	-2.613656	-1.014065	1.272118
C	-3.962402	-2.687798	1.007448
H	2.138943	-2.526852	2.942152
H	1.765050	-0.854946	2.483654
C	1.191195	-1.362794	4.511472
H	1.713049	-4.426433	-1.194084
H	1.854726	-2.658483	-1.183462
C	3.175886	-3.647689	0.208443
H	-4.793881	-2.217085	1.543331
H	-4.102737	-2.512711	-0.063582
H	-3.983647	-3.766540	1.191491
H	0.813535	-2.226968	5.066910
H	2.137596	-1.042198	4.961627
H	0.464362	-0.549210	4.604686
H	3.183357	-4.530685	0.856289
H	4.009802	-3.713360	-0.499124
H	3.331655	-2.750789	0.812730

INT5

C	1.191857	-1.817989	2.730537
O	0.822408	-3.031811	3.200111
O	1.170003	-0.807902	3.415723
C	0.378771	-3.051485	4.563952
H	0.120812	-4.089904	4.774613
H	1.172486	-2.706796	5.233607
H	-0.495054	-2.405439	4.692937
C	1.593112	-1.903590	1.311616
H	1.499370	-2.872089	0.834526
C	2.008759	-0.810384	0.655595
H	2.063748	0.131171	1.199182
C	2.327602	-0.695996	-0.826121
C	3.720845	-0.062947	-0.969361
C	4.828341	-0.633921	-0.327127
C	3.908565	1.077030	-1.755954
C	6.099249	-0.076631	-0.469475
H	4.691149	-1.515253	0.295161
C	5.181428	1.634192	-1.902713
H	3.047309	1.512748	-2.250209
C	6.280511	1.061129	-1.260356
H	6.947270	-0.528976	0.038616
H	5.314203	2.518566	-2.521598
H	7.270050	1.496633	-1.372974
C	2.288449	-2.042842	-1.570862
H	3.034404	-2.748021	-1.187431
H	1.290987	-2.482790	-1.489695
H	2.499113	-1.856139	-2.627740
Cu	-0.309263	0.179730	-0.551050
O	1.347675	0.165204	-1.373880
S	-2.429202	-1.459087	-0.562545
O	-2.355733	0.035163	-0.276924
O	-1.008209	-1.900540	-0.771472
O	-3.285476	-2.243791	0.316756
C	-3.185672	-1.514393	-2.263483
F	-3.230756	-2.772538	-2.701309
F	-4.421757	-1.010262	-2.223391
F	-2.440130	-0.780507	-3.101151
N	-0.119536	1.973794	0.188176
H	0.582010	2.515202	-0.307881
C	-1.052622	2.673926	0.802795
N	-1.786913	2.110893	1.803165
N	-1.321790	3.980779	0.480680
C	-3.234832	2.295938	1.881905
H	-3.560172	3.055261	1.170335
H	-3.727456	1.350622	1.625319
H	-3.533649	2.597411	2.892865
C	-1.279751	0.947559	2.527270
H	-1.659232	0.015551	2.093435
H	-0.192015	0.928184	2.509403
H	-1.616209	1.012299	3.567817
C	-0.908258	4.516728	-0.809015
H	-0.934977	3.729194	-1.565714
H	-1.606658	5.306676	-1.104523
H	0.102810	4.949637	-0.776306
C	-1.687116	4.971447	1.488968

H	-0.934955	5.771149	1.513250
H	-2.663398	5.421177	1.269296
H	-1.725416	4.501437	2.471858

INT6

C	0.020284	-1.867216	2.987367
O	-1.276164	-2.240648	3.012632
O	0.512435	-1.028818	3.712976
C	-2.109795	-1.515197	3.949473
H	-3.084593	-2.000303	3.901795
H	-1.691309	-1.584321	4.955664
H	-2.177363	-0.470442	3.639591
C	0.719558	-2.562657	1.873954
H	0.215159	-3.423928	1.443908
C	1.817990	-2.021597	1.335778
H	2.237971	-1.142395	1.811531
C	2.380224	-2.414602	-0.021761
C	3.675952	-1.688555	-0.362952
C	4.604722	-1.363636	0.632760
C	3.972311	-1.373870	-1.697376
C	5.791954	-0.704478	0.306743
H	4.413860	-1.625086	1.669888
C	5.158032	-0.711518	-2.022054
H	3.267801	-1.638928	-2.478753
C	6.068102	-0.366560	-1.019342
H	6.500697	-0.457954	1.091846
H	5.373426	-0.473872	-3.060144
H	6.991491	0.146460	-1.271622
C	2.554903	-3.928160	-0.200001
H	3.317211	-4.298187	0.491069
H	1.623349	-4.470431	0.000467
H	2.878401	-4.142946	-1.222265
Cu	0.098107	-0.207920	-0.445881
O	1.377279	-1.948340	-1.005420
S	-1.983199	1.810245	0.493745
O	-2.094493	0.767709	1.534086
O	-1.646285	3.170665	0.908417
O	-1.154772	1.322289	-0.702877
C	-3.682284	1.944522	-0.268759
F	-3.611251	2.520897	-1.471677
F	-4.210602	0.712648	-0.415292
F	-4.472814	2.656322	0.526039
N	1.712588	0.814901	-0.322027
H	2.423518	0.444229	-0.946314
C	1.998538	2.062790	0.054965
N	1.488012	2.593549	1.186201
N	2.843930	2.831045	-0.699424
C	1.286156	4.033172	1.366478
H	1.365309	4.543357	0.406463
H	0.278348	4.187296	1.755085
H	2.017047	4.453632	2.067682
C	1.084285	1.767525	2.324227
H	0.008060	1.825404	2.493532
H	1.355570	0.730838	2.152465
H	1.600452	2.122834	3.223853
C	3.011826	2.562167	-2.124549
H	2.076728	2.185116	-2.544241
H	3.266211	3.499454	-2.628994
H	3.815858	1.836848	-2.314422
C	3.903436	3.639951	-0.093668
H	4.876894	3.282330	-0.451930
H	3.798763	4.698382	-0.356863
H	3.883385	3.538263	0.991091
N	-1.305580	-1.573467	-0.480044
H	-1.465314	-2.002397	0.428227
C	-2.400043	-1.598770	-1.240577
N	-2.345158	-1.187481	-2.539922
N	-3.581037	-2.072684	-0.762008
C	-3.440371	-0.426939	-3.156109
H	-4.323606	-0.435849	-2.524398
H	-3.128881	0.617303	-3.275846
H	-3.686228	-0.845586	-4.137228
C	-1.063908	-0.993624	-3.208525
H	-0.671632	0.020528	-3.043014
H	-0.331936	-1.726050	-2.868029
H	-1.210548	-1.128322	-4.284556
C	-3.854470	-2.075069	0.675846
H	-3.354188	-1.231852	1.153775
H	-4.932474	-1.957557	0.817984
H	-3.541040	-3.014765	1.150901
C	-4.527453	-2.823284	-1.587311
H	-4.706626	-3.800888	-1.124261
H	-5.487019	-2.299883	-1.670362
H	-4.116905	-2.981915	-2.584690

H	0.609255	-2.551737	-0.965452

INT7

C	0.526132	-3.125348	-1.078588
O	-0.102400	-4.004089	-1.871058
O	0.720468	-3.305448	0.114329
C	-0.575508	-5.205216	-1.226939
H	-1.013084	-5.806958	-2.022879
H	0.253339	-5.734141	-0.750389
H	-1.325930	-4.951964	-0.474233
C	0.917025	-1.906123	-1.831060
H	0.535192	-1.807351	-2.841303
C	1.684398	-0.983906	-1.241916
H	2.028268	-1.180705	-0.232506
C	2.073876	0.373262	-1.768087
C	3.587791	0.575298	-1.769188
C	4.478159	-0.488932	-1.586857
C	4.101440	1.871238	-1.927449
C	5.856643	-0.261782	-1.552639
H	4.102924	-1.502557	-1.482098
C	5.476201	2.098994	-1.888042
H	3.419665	2.705521	-2.059317
C	6.358840	1.032184	-1.696563
H	6.535824	-1.098826	-1.417638
H	5.859202	3.108453	-2.008046
H	7.430229	1.208427	-1.668506
C	1.438825	0.721839	-3.114947
H	1.811463	0.052231	-3.895649
H	0.351384	0.626087	-3.053930
H	1.691982	1.747398	-3.394400
Cu	-0.385598	0.058450	0.744237
O	1.642786	1.340060	-0.746525
O	0.230781	1.285068	-0.572647
S	-2.977673	-0.711981	0.899322
O	-1.974869	-1.152764	1.912242
O	-4.241895	-0.126123	1.342791
O	-2.208067	0.136164	-0.113365
C	-3.417118	-2.223070	-0.103429
F	-4.359867	-1.894948	-0.992969
F	-2.330165	-2.646266	-0.761368
F	-3.859118	-3.188410	0.692652
N	0.955894	-0.478942	2.013888
H	0.746763	-1.419502	2.339622
C	2.207035	-0.105306	2.247140
N	2.553877	1.208479	2.183966
N	3.184604	-1.000361	2.576278
C	3.831507	1.653712	1.623701
H	4.388622	0.806075	1.224605
H	3.631496	2.333928	0.791951
H	4.438422	2.170471	2.376279
C	1.547328	2.250233	2.329651
H	1.169945	2.568856	1.353789
H	0.716384	1.877305	2.931534
H	2.001463	3.107420	2.838589
C	3.027639	-2.432283	2.323013
H	2.358857	-2.615687	1.481841
H	4.010427	-2.853120	2.087385
H	2.636646	-2.953758	3.208192
C	4.259859	-0.661495	3.511473
H	4.263058	-1.389805	4.330823
H	5.238983	-0.687317	3.019640
H	4.096849	0.330075	3.933231
N	-1.998293	2.487965	-1.898536
H	-2.388841	2.148230	-2.765744
C	-2.825370	2.701324	-0.854107
N	-2.319187	3.349994	0.220150
N	-4.117880	2.347609	-0.904781
C	-2.609927	2.918820	1.591160
H	-3.376741	2.148300	1.605565
H	-1.696200	2.492583	2.026654
H	-2.927718	3.769187	2.202763
C	-1.125643	4.190586	0.073759
H	-0.211809	3.593482	0.143517
H	-1.148147	4.699893	-0.890878
H	-1.139903	4.937840	0.871899
C	-4.593780	1.262944	-1.767187
H	-3.768116	0.604911	-2.034707
H	-5.316972	0.669156	-1.202699
H	-5.078649	1.659387	-2.667110
C	-5.154734	2.992493	-0.095389
H	-6.010554	3.211613	-0.741705
H	-5.480354	2.328631	0.712180
H	-4.780342	3.928752	0.318516
H	-1.057819	2.147809	-1.659645

INT8

C	-3.031762	-0.195610	-0.209301
O	-4.001610	-0.558439	0.665704
O	-3.188088	-0.182612	-1.414490
C	-5.252548	-0.926654	0.065517
H	-5.912456	-1.185626	0.894417
H	-5.664251	-0.091617	-0.509053
H	-5.121363	-1.781435	-0.604794
C	-1.789026	0.167124	0.514154
H	-1.812246	0.076078	1.595173
C	-0.715169	0.584615	-0.164109
H	-0.774643	0.649567	-1.247191
C	0.616844	0.983413	0.423592
C	1.697469	-0.081138	0.184526
C	1.387355	-1.433252	0.006870
C	3.045110	0.311409	0.182677
C	2.403460	-2.375576	-0.170763
H	0.349716	-1.752073	0.005110
C	4.059290	-0.628521	-0.002766
H	3.291932	1.358854	0.336941
C	3.740873	-1.977225	-0.180316
H	2.145744	-3.422266	-0.308407
H	5.097680	-0.307688	-0.004742
H	4.529389	-2.710640	-0.324716
C	0.553042	1.346366	1.913512
H	1.528778	1.711490	2.244519
H	-0.198317	2.122252	2.085407
H	0.301829	0.461100	2.504206
O	1.149873	2.068672	-1.615519
O	1.027256	2.238747	-0.181077
H	2.049146	1.691364	-1.685387

INTA1

C	-2.956555	-0.396436	-0.210778
O	-3.962629	-0.496475	0.688502
O	-3.057095	-0.729818	-1.374968
C	-5.192814	-1.025571	0.168382
H	-5.886028	-1.039793	1.010071
H	-5.574293	-0.389472	-0.635649
H	-5.040776	-2.035195	-0.224514
C	-1.741781	0.167302	0.429619
H	-1.804153	0.387208	1.489899
C	-0.643215	0.388109	-0.297138
H	-0.663398	0.143962	-1.357401
C	0.664265	0.932066	0.212887
C	1.800329	-0.075130	0.064094
C	1.552927	-1.451310	0.127896
C	3.120669	0.375418	-0.077589
C	2.607117	-2.363549	0.049841
H	0.535117	-1.812438	0.239772
C	4.171199	-0.537724	-0.163047
H	3.323053	1.439987	-0.133785
C	3.918292	-1.910055	-0.098401
H	2.399543	-3.428875	0.100640
H	5.188893	-0.175457	-0.280369
H	4.738102	-2.620133	-0.164030
C	0.618405	1.519871	1.622770
H	1.594506	1.944544	1.872152
H	-0.141324	2.302974	1.679998
H	0.393829	0.732769	2.348180
O	0.160848	3.031388	-0.699230
O	1.036864	2.046403	-0.728825

INTA2

C	-2.195597	0.231563	-0.164193
O	-2.983089	-0.617935	-0.862442
O	-2.340597	0.465624	1.032505
C	-4.012162	-1.260724	-0.095202
H	-4.549152	-1.896788	-0.799439
H	-3.573773	-1.860132	0.708661
H	-4.684682	-0.519366	0.346562
C	-1.144644	0.813159	-0.973945
H	-0.967309	0.475695	-1.988113
C	-0.233277	1.779917	-0.337569
H	-0.794181	2.524280	0.245354
C	0.998920	1.261220	0.470154
C	1.335029	-0.202736	0.252195
C	0.622941	-1.198086	0.939764
C	2.335399	-0.577291	-0.649955
C	0.914693	-2.545279	0.727914
H	-0.175742	-0.921524	1.621453
C	2.630255	-1.927471	-0.853601
H	2.874773	0.194842	-1.186827

C	1.922732	-2.914744	-0.166557
H	0.353822	-3.307025	1.263123
H	3.413195	-2.205743	-1.554155
H	2.151315	-3.964761	-0.328231
C	1.052484	1.688620	1.927195
H	2.026133	1.438823	2.361056
H	0.893584	2.768174	2.003515
H	0.272002	1.177093	2.498603
O	0.676035	2.410101	-1.267750
O	1.822572	2.143154	-0.358408

INTB1

C	1.755806	-0.329994	2.469158
O	1.585998	0.532756	3.521190
O	2.050550	-1.509656	2.656382
C	1.708139	-0.059277	4.820705
H	1.531107	0.750785	5.530427
H	0.969058	-0.854820	4.956341
H	2.706956	-0.481720	4.964589
C	1.492749	0.304701	1.188534
H	1.511700	1.386333	1.240534
C	2.176289	-0.312139	0.015827
H	2.591407	-1.284174	0.290149
C	3.107089	0.521843	-0.906223
C	3.400970	1.969342	-0.537285
H	3.886995	2.466393	-1.383833
H	2.484085	2.510903	-0.293400
H	4.082057	2.014202	0.319129
C	4.371517	-0.220233	-1.298205
C	5.305310	-0.555496	-0.308349
C	4.627813	-0.564741	-2.627760
C	6.484111	-1.219746	-0.645508
H	5.101409	-0.307788	0.731603
C	5.810660	-1.227273	-2.965225
H	3.890632	-0.324452	-3.386664
C	6.741749	-1.554420	-1.977972
H	7.198649	-1.479884	0.131023
H	6.000586	-1.492677	-4.001977
H	7.659591	-2.072820	-2.242153
Cu	-0.561736	-0.056879	0.569070
S	-2.818992	-0.063696	-1.967379
O	-2.137743	1.125949	-2.524546
O	-2.063254	-0.724367	-0.841587
O	-3.414927	-1.018263	-2.904588
C	-4.273060	0.659741	-1.048820
F	-5.066746	-0.318131	-0.567526
F	-5.004170	1.443569	-1.846423
F	-3.855181	1.396099	0.002964
N	-0.553734	-1.863323	1.331252
H	0.392576	-2.148082	1.579926
C	-1.275526	-2.875274	0.875566
N	-2.633567	-2.849144	0.947932
N	-0.685855	-3.989280	0.338885
C	-3.467237	-3.357563	-0.144594
H	-2.839179	-3.752720	-0.941992
H	-4.049451	-2.535618	-0.566213
H	-4.147065	-4.140903	0.213034
C	-3.278528	-1.862127	1.806710
H	-3.394512	-0.902512	1.293323
H	-2.680886	-1.714525	2.707379
H	-4.269508	-2.238580	2.081876
C	0.668041	-3.896906	-0.199640
H	0.817903	-2.919139	-0.664287
H	0.788609	-4.666194	-0.969085
H	1.436694	-4.053679	0.571267
C	-1.211377	-5.333757	0.540286
H	-0.454029	-5.955905	1.035704
H	-1.480475	-5.808372	-0.411834
H	-2.095851	-5.293516	1.177076
N	-0.842269	1.777755	-0.039071
H	-1.125040	1.769612	-1.021151
C	-0.388353	2.925315	0.383328
N	-0.310291	3.188995	1.729914
N	0.042878	3.912858	-0.478028
C	0.837362	3.870404	2.323324
H	1.498453	4.240159	1.538453
H	1.397759	3.166915	2.952939
H	0.514336	4.716598	2.943282
C	-1.105518	2.393851	2.660267
H	-0.558147	1.510035	3.008467
H	-2.027284	2.076874	2.170146
H	-1.352661	3.018010	3.527365
C	0.251669	3.565513	-1.885741
H	0.740716	2.592108	-1.968697

H	0.901049	4.325469	-2.331651
H	-0.692027	3.532736	-2.447602
C	-0.300125	5.314597	-0.243317
H	-1.092568	5.632635	-0.935746
H	0.574514	5.956618	-0.400983
H	-0.661124	5.449885	0.776219
O	1.286849	-0.490414	-1.162078
O	2.094352	0.471920	-1.955475

INTB2

C	-1.393646	-3.085790	-0.136661
O	-2.730507	-3.220716	-0.405252
O	-0.568659	-3.828308	-0.657933
C	-3.057780	-4.264534	-1.332939
H	-4.146299	-4.254693	-1.416089
H	-2.599412	-4.075781	-2.309007
H	-2.711611	-5.233853	-0.964650
C	-1.102987	-1.962820	0.765471
H	-1.982853	-1.616875	1.307393
C	0.063062	-2.258440	1.662845
H	0.817518	-2.828903	1.112432
C	0.737052	-1.233273	2.614065
C	-0.081794	0.012537	2.921980
H	-0.119869	0.686227	2.063129
H	-1.098249	-0.292807	3.186049
H	0.355920	0.550160	3.768866
C	2.212030	-0.992651	2.360317
C	3.114049	-2.056323	2.531787
C	2.701536	0.239329	1.910819
C	4.469110	-1.889808	2.248664
H	2.742294	-3.006185	2.902394
C	4.054939	0.400328	1.604457
H	2.034892	1.075976	1.772845
C	4.943428	-0.661564	1.775374
H	5.155606	-2.719648	2.396964
H	4.395396	1.358825	1.223602
H	5.998624	-0.535467	1.545716
Cu	-0.637418	-0.359606	-0.517614
S	1.098907	2.688579	-0.624062
O	2.551589	2.905965	-0.648898
O	0.713060	1.303433	-0.185408
O	0.306554	3.196417	-1.759557
C	0.499724	3.698090	0.825036
F	-0.849755	3.579196	0.952962
F	0.783605	4.990914	0.668329
F	1.048257	3.268418	1.973232
N	0.612022	-1.337465	-1.632050
H	0.786956	-2.290297	-1.320024
C	1.591139	-0.821733	-2.357485
N	1.347930	0.215104	-3.203375
N	2.867156	-1.313926	-2.298715
C	2.350951	1.239744	-3.505005
H	3.151680	1.205308	-2.765875
H	1.877253	2.220577	-3.431222
H	2.767159	1.115603	-4.513122
C	-0.018289	0.519826	-3.620061
H	-0.459637	1.293675	-2.982334
H	-0.620504	-0.388767	-3.572981
H	0.004540	0.888557	-4.651808
C	3.312872	-2.027409	-1.106975
H	2.802732	-1.635368	-0.227957
H	4.384901	-1.858481	-0.973297
H	3.135340	-3.110536	-1.181351
C	3.706475	-1.458264	-3.481849
H	4.026960	-2.504170	-3.574165
H	4.601058	-0.825427	-3.425590
H	3.142045	-1.195232	-4.377249
N	-2.087910	0.881021	-0.066927
H	-1.727117	1.725539	0.365261
C	-3.386619	0.739571	0.050109
N	-4.068477	-0.103343	-0.783793
N	-4.125968	1.417840	0.992721
C	-5.149795	-0.960721	-0.302462
H	-5.404787	-0.698200	0.724362
H	-4.818054	-2.004086	-0.318006
H	-6.042150	-0.851274	-0.931238
C	-3.479589	-0.492866	-2.060053
H	-2.871408	-1.398726	-1.967946
H	-2.854238	0.318557	-2.435211
H	-4.290450	-0.683210	-2.772633
C	-3.457170	2.008432	2.146847
H	-2.629646	1.371374	2.463766
H	-4.177198	2.078927	2.968469
H	-3.068817	3.014668	1.937157

C	-5.427660	1.997576	0.670491
H	-5.360568	3.094338	0.674105
H	-6.184405	1.694718	1.404361
H	-5.745925	1.675838	-0.321212
O	0.555600	-2.206884	3.699031
O	-0.355579	-3.018200	2.845206

INTB3

C	4.261365	-1.361483	-1.395353
O	5.210331	-2.198900	-1.910976
O	4.407752	-0.137020	-1.427118
C	6.350961	-1.547905	-2.474022
H	7.007682	-2.348169	-2.821036
H	6.063324	-0.902760	-3.310501
H	6.863395	-0.932981	-1.726235
C	3.161655	-2.093851	-0.805290
H	3.229951	-3.170617	-0.701343
C	2.081593	-1.383859	-0.363192
H	2.089668	-0.314916	-0.588079
Cu	-0.359262	-0.600915	0.786306
S	-1.989848	1.160551	-0.978930
O	-0.766675	0.738110	-1.694006
O	-1.925890	0.812284	0.498437
O	-3.284534	0.863892	-1.605959
C	-1.882794	3.017443	-0.903078
F	-2.914206	3.519817	-0.217788
F	-1.873347	3.538236	-2.131390
F	-0.739804	3.380962	-0.277316
N	-1.604050	-2.079991	0.459300
H	-1.157290	-2.746492	-0.164819
C	-2.909269	-1.964943	0.276750
N	-3.684630	-1.419543	1.258726
N	-3.523736	-2.392950	-0.863548
C	-4.752887	-0.468571	0.940087
H	-4.839706	-0.344560	-0.137201
H	-4.486514	0.510400	1.352648
H	-5.708472	-0.796257	1.366982
C	-3.130405	-1.273833	2.598958
H	-2.559091	-0.340180	2.692316
H	-2.473918	-2.115758	2.822102
H	-3.957434	-1.253887	3.316877
C	-2.791215	-2.435634	-2.125376
H	-1.953740	-1.736585	-2.097621
H	-3.462449	-2.119253	-2.930113
H	-2.423628	-3.446762	-2.351192
C	-4.885888	-2.905836	-0.893425
H	-4.894298	-3.881951	-1.395060
H	-5.560172	-2.232175	-1.437620
H	-5.256207	-3.034792	0.124393
N	0.698688	0.930826	1.385749
H	0.279546	1.781219	1.021478
C	2.018034	1.033031	1.495796
N	2.691885	0.169839	2.303734
N	2.716534	2.001890	0.833936
C	4.035275	-0.320734	1.984406
H	4.349799	0.033401	1.003724
H	4.006950	-1.413509	1.936191
H	4.758727	-0.013734	2.749865
C	1.950719	-0.650450	3.256872
H	1.606513	-1.578811	2.782448
H	1.084951	-0.097394	3.623382
H	2.612209	-0.894692	4.094690
C	2.314774	2.445918	-0.502342
H	1.436244	1.897223	-0.842069
H	3.136002	2.242328	-1.198542
H	2.081860	3.517832	-0.501937
C	3.952576	2.589032	1.331307
H	3.884613	3.680594	1.242686
H	4.823097	2.249797	0.755943
H	4.092017	2.337936	2.383778
O	1.061384	-1.849413	0.296428

TS1

C	-0.763974	1.525154	-2.419203
O	-1.334428	2.496102	-3.156822
O	-1.488828	0.556806	-2.054165
C	-2.729778	2.364813	-3.451330
H	-2.983796	3.240060	-4.049463
H	-3.317890	2.347450	-2.529415
H	-2.922064	1.448617	-4.017918
C	0.624778	1.763814	-2.195601
H	1.007206	2.658153	-2.673065
C	1.455042	0.939459	-1.471170
H	1.011191	0.063258	-1.003676

C	2.838637	1.169092	-1.163460	O	0.852703	0.401441	2.639308
H	2.646918	1.854327	-0.034330	C	0.060717	-1.981939	3.486948
C	3.586515	2.176845	-2.032297	F	1.293851	-2.455183	3.251812
H	4.590009	2.353643	-1.634738	F	-0.043950	-1.632897	4.763983
H	3.067710	3.141647	-2.050623	F	-0.827383	-2.954398	3.217825
H	3.688839	1.840200	-3.072624	N	-0.310836	-2.058314	-1.802068
N	2.281606	2.561998	1.098454	H	-0.104006	-1.664295	-2.714488
H	1.725576	1.940145	1.684372	C	0.499386	-3.059332	-1.458598
C	1.782010	3.767818	0.956725	N	0.156202	-3.881055	-0.429952
N	2.575896	4.773859	0.469156	N	1.653857	-3.314249	-2.129712
N	0.475867	4.102622	1.249490	C	1.143889	-4.387301	0.525275
C	2.067255	5.815812	-0.414150	H	2.120425	-3.950240	0.322389
H	1.000917	5.670964	-0.590254	H	0.844977	-4.086534	1.531880
H	2.589363	5.766718	-1.379641	H	1.211469	-5.480934	0.488570
H	2.228107	6.815261	0.009089	C	-1.233758	-4.023469	-0.007821
C	4.027953	4.677898	0.551079	H	-1.467769	-3.356637	0.828421
H	4.467012	4.376149	-0.408251	H	-1.897853	-3.807542	-0.846207
H	4.301044	3.941611	1.306553	H	-1.395252	-5.058154	0.313432
H	4.436489	5.658246	0.823703	C	2.307668	-2.301528	-2.954182
C	-0.543735	3.062951	1.292226	H	2.026039	-1.300752	-2.632003
H	-0.324991	2.301373	0.540071	H	3.389202	-2.390179	-2.825760
H	-1.509317	3.521361	1.047491	H	2.059166	-2.433347	-4.016478
H	-0.623350	2.579106	2.273471	C	2.224661	-4.655450	-2.252131
C	0.143302	5.326855	1.975261	H	2.436463	-4.852268	-3.309173
H	-0.225469	5.080019	2.980171	H	3.160524	-4.739456	-1.688223
H	-0.633828	5.896622	1.450734	H	1.516211	-5.403974	-1.898033
H	1.029511	5.952698	2.081064	N	-2.979962	-0.421132	0.281756
C	3.611560	-0.039404	-0.702715	H	-2.804333	-0.281018	1.277040
C	4.799145	-0.451273	-1.329174	C	-4.152172	0.025386	-0.127936
C	3.148144	-0.791905	0.389999	N	-4.660475	-0.347290	-1.337645
C	5.480032	-1.593713	-0.899251	N	-4.910941	0.867096	0.645276
H	5.191951	0.110988	-2.170873	C	-5.534947	0.516986	-2.126551
C	3.831537	-1.925823	0.826984	H	-5.629441	1.493009	-1.650258
H	2.259820	-0.472571	0.919962	H	-5.100641	0.655152	-3.123167
C	4.997499	-2.342228	0.177182	H	-6.532872	0.078502	-2.245385
H	6.394902	-1.893522	-1.403941	C	-4.130244	-1.504501	-2.048589
H	3.460840	-2.463093	1.695866	H	-3.306041	-1.225131	-2.717347
H	5.538501	-3.218812	0.522951	H	-3.775909	-2.248606	-1.332911
Cu	-1.376921	-0.911286	-0.669380	H	-4.933393	-1.949246	-2.644739
S	-0.269260	-0.513041	2.393194	C	-4.273822	1.622636	1.723540
O	-1.615718	-0.035750	2.766265	H	-3.275457	1.939545	1.419211
O	-0.214965	-1.161492	1.018692	H	-4.881264	2.509519	1.926854

H	-4.183738	1.038154	2.648786
C	-6.353488	0.662907	0.811552
H	-6.565726	0.387490	1.852248
H	-6.910601	1.575795	0.572873
H	-6.700632	-0.146168	0.168966

TS2

C	1.686966	1.188589	2.790066
O	0.722166	1.912381	3.415174
O	2.278554	0.269359	3.336723
C	0.433900	1.503770	4.761449
H	-0.356487	2.171023	5.107261
H	0.095036	0.463770	4.781717
H	1.322841	1.598885	5.391106
C	1.903975	1.644987	1.410925
H	1.185596	2.338504	0.992974
C	2.997413	1.197302	0.732807
H	3.661074	0.533072	1.278890
C	3.317902	1.492772	-0.632816
C	2.468434	2.488525	-1.364968
H	2.724363	2.546809	-2.424274
H	1.416593	2.180541	-1.295651
H	2.552018	3.489119	-0.921004
C	4.627043	1.083952	-1.163313
C	5.311624	1.870707	-2.110782
C	5.233151	-0.120908	-0.746011
C	6.552105	1.477438	-2.608867
H	4.883252	2.810685	-2.442862
C	6.465547	-0.517897	-1.254165
H	4.715427	-0.767375	-0.045020
C	7.134847	0.280997	-2.186145
H	7.064656	2.108531	-3.329755
H	6.903525	-1.457417	-0.928114
H	8.098463	-0.028497	-2.581005
Cu	-0.328890	-0.165501	0.369043
S	-2.565040	-1.037281	-2.200022
O	-2.236132	0.304718	-2.744018
O	-1.663630	-1.480796	-1.090133
O	-2.907360	-2.084658	-3.168059
C	-4.164539	-0.733595	-1.287101
F	-4.527314	-1.826658	-0.588318
F	-5.156246	-0.415884	-2.126801
F	-4.042912	0.284648	-0.396233
N	0.561778	-1.530859	1.372141
H	1.489214	-1.290162	1.714028
C	0.414913	-2.823423	1.151009
N	-0.824578	-3.391322	1.097050
N	1.491631	-3.660745	0.983070
C	-1.176372	-4.362905	0.058772
H	-0.283601	-4.662353	-0.490233
H	-1.867924	-3.892919	-0.648010
H	-1.641559	-5.251290	0.503776
C	-1.967554	-2.724492	1.711126
H	-2.526183	-2.144057	0.971630
H	-1.618383	-2.063614	2.505708
H	-2.628249	-3.489559	2.137408
C	2.786707	-3.115050	0.598824
H	2.648376	-2.236158	-0.033465
H	3.331696	-3.871536	0.023746
H	3.398640	-2.843996	1.473909
C	1.506963	-5.023028	1.502173
H	2.309039	-5.129449	2.245746
H	1.679801	-5.753223	0.701372
H	0.554762	-5.246110	1.984014
N	-1.234112	1.404946	-0.270410
H	-1.332798	1.313874	-1.284666
C	-2.013062	2.316900	0.233010
N	-2.368418	2.319647	1.564661
N	-2.534883	3.356374	-0.524434
C	-2.270738	3.539619	2.358020
H	-2.200404	4.403168	1.695842
H	-1.373679	3.507566	2.992960
H	-3.153955	3.655644	2.998995
C	-2.364814	1.078048	2.323045
H	-1.375736	0.830483	2.725787
H	-2.708361	0.261938	1.686645
H	-3.066304	1.185119	3.159532
C	-1.990998	3.559731	-1.866626
H	-0.898615	3.524898	-1.830256
H	-2.295657	4.551654	-2.214755
H	-2.345993	2.803515	-2.580160
C	-3.974850	3.620272	-0.435539
H	-4.529103	2.985270	-1.141956
H	-4.171447	4.671371	-0.671785

H	-4.334102	3.408932	0.571056
O	1.013114	-0.043447	-1.411779
O	2.248237	-0.280024	-1.298502

TS2'

C	-1.098714	-0.635424	2.270618
O	-2.365008	-0.988030	2.514056
O	-0.603405	0.424309	2.632352
C	-3.198825	0.038756	3.077259
H	-4.175699	-0.423980	3.209975
H	-2.792176	0.384280	4.031840
H	-3.266860	0.871812	2.374192
C	-0.380910	-1.691432	1.517298
H	-0.971878	-2.536485	1.189778
C	0.943502	-1.535446	1.260088
H	1.384409	-0.601689	1.595040
C	1.740205	-2.410971	0.455118
C	3.182423	-2.129991	0.307029
C	3.889638	-1.360964	1.254002
C	3.889750	-2.599076	-0.819827
C	5.244801	-1.080909	1.083744
H	3.386497	-1.007999	2.147998
C	5.240005	-2.305684	-0.995268
H	3.365169	-3.165254	-1.581854
C	5.926640	-1.546745	-0.043319
H	5.771547	-0.506871	1.841494
H	5.757003	-2.668579	-1.879260
H	6.982127	-1.326194	-0.175569
C	1.200664	-3.776964	0.117723
H	1.135587	-4.381746	1.031855
H	0.194425	-3.702474	-0.306511
H	1.836108	-4.311054	-0.590503
Cu	-0.691165	0.996091	-0.519546
O	1.187574	-1.600514	-1.451075
O	-0.049497	-1.438819	-1.539358
S	-3.222441	-0.459879	-1.011699
O	-3.119488	-0.313576	-2.466325
O	-2.991913	-1.780001	-0.414119
O	-2.519200	0.676398	-0.240195
C	-4.978481	-0.015646	-0.584494
F	-5.164078	-0.038985	0.746953
F	-5.276693	1.209503	-1.033106
F	-5.812800	-0.898027	-1.145913
N	1.173507	1.226399	-0.603253
H	1.634098	0.382852	-0.934935
C	2.019588	2.191611	-0.337383
N	1.655810	3.289904	0.398210
N	3.323921	2.160688	-0.789811
C	2.076354	4.630268	-0.005503
H	2.828779	4.570944	-0.791931
H	1.213662	5.191533	-0.391429
H	2.491854	5.180928	0.846984
C	0.465400	3.255149	1.242438
H	-0.446314	3.503137	0.678380
H	0.342004	2.271949	1.697654
H	0.590361	3.998791	2.037175
C	3.691983	1.220899	-1.843948
H	2.902006	1.176273	-2.597798
H	4.608042	1.580484	-2.323087
H	3.881227	0.209076	-1.458969
C	4.429314	2.553232	0.084281
H	4.987541	1.662747	0.403498
H	5.116077	3.228488	-0.440040
H	4.044204	3.056988	0.970770

α-TS2

C	-1.359690	1.098191	-2.106106
O	-0.366332	1.939585	-2.453272
O	-1.327440	-0.105649	-2.324794
C	0.771382	1.319260	-3.086089
H	1.395633	2.144048	-3.431084
H	0.452634	0.699763	-3.928076
H	1.320215	0.699869	-2.374779
C	-2.444009	1.801546	-1.405860
H	-2.360735	2.877345	-1.339710
C	-3.647506	1.111033	-1.158787
H	-3.637025	0.073631	-1.473850
C	-4.750059	1.581281	-0.479936
C	-4.788316	2.972148	0.104571
H	-4.634988	2.943615	1.191425
H	-5.757139	3.451737	-0.076381
H	-4.006318	3.612714	-0.306209
C	-5.906993	0.699747	-0.240219
C	-6.805249	0.958849	0.816821

C	-6.163393	-0.433571	-1.045184
C	-7.885389	0.116172	1.073220
H	-6.647215	1.817367	1.460811
C	-7.243888	-1.271942	-0.790061
H	-5.527609	-0.641825	-1.899621
C	-8.111102	-1.006024	0.274247
H	-8.552326	0.337932	1.902248
H	-7.418393	-2.130476	-1.433296
H	-8.956074	-1.660112	0.470701
O	-0.238466	1.863446	0.576386
O	-1.532055	1.683368	0.548755
Cu	0.915120	0.180766	0.474079
S	3.110122	-1.554883	-1.415617
O	3.586898	-0.221159	-1.862061
O	1.849567	-1.485748	-0.591423
O	3.131971	-2.644627	-2.391680
C	4.371334	-2.032624	-0.128954
F	4.099372	-3.246380	0.386244
F	5.601961	-2.057719	-0.648251
F	4.358680	-1.145100	0.889692
N	-0.585277	-0.933142	0.977771
H	-1.466487	-0.449325	0.813998
C	-0.660684	-2.241571	1.049682
N	0.368501	-2.974785	1.581760
N	-1.762074	-2.946423	0.632433
C	0.851402	-4.179251	0.903525
H	0.157015	-4.467813	0.114908
H	1.821682	-3.973415	0.442634
H	0.954376	-5.007592	1.615705
C	1.353470	-2.328542	2.439077
H	2.194498	-1.924166	1.866526
H	0.878141	-1.518447	2.995335
H	1.739048	-3.072754	3.145974
C	-2.717375	-2.337590	-0.283877
H	-2.200026	-1.669405	-0.974673
H	-3.199150	-3.132184	-0.864028
H	-3.505727	-1.781442	0.247116
C	-2.235139	-4.130566	1.339363
H	-3.239757	-3.945167	1.744550
H	-2.290032	-4.997439	0.668541
H	-1.563883	-4.365171	2.165744
N	2.518636	1.275173	0.383625
H	3.157621	0.968212	-0.350516
C	2.705779	2.500695	0.798670
N	2.150383	2.947940	1.976293
N	3.471988	3.407314	0.102260
C	1.426997	4.217778	2.015764
H	1.687184	4.825216	1.148729
H	0.346524	4.026194	1.989194
H	1.675412	4.772277	2.928953
C	1.723944	1.986464	2.985134
H	0.678166	1.684902	2.845213
H	2.356807	1.098830	2.934926
H	1.830729	2.449213	3.973611
C	3.841641	3.117066	-1.278683
H	3.016084	2.622317	-1.790871
H	4.059741	4.061450	-1.787558
H	4.726095	2.467763	-1.347610
C	4.339057	4.362992	0.784817
H	5.393641	4.105104	0.612516
H	4.168244	5.380803	0.412851
H	4.147314	4.339615	1.857397

TS3

C	-6.602776	0.269336	0.475899
O	-7.280660	0.614488	-0.650380
O	-7.154234	-0.136880	1.482037
C	-8.704636	0.471184	-0.566636
H	-9.091258	0.785444	-1.537321
H	-8.978212	-0.568587	-0.362186
H	-9.111036	1.100718	0.230917
C	-5.149591	0.453683	0.286118
H	-4.804990	0.821465	-0.673412
C	-4.296066	0.178195	1.279216
H	-4.694960	-0.174476	2.229496
C	-2.789550	0.368573	1.220583
Cu	0.527524	-0.481056	-0.252455
O	-2.446921	0.578677	-0.127447
O	-0.659428	0.962065	-0.131605
S	2.904679	-2.309550	0.943662
O	3.323382	-2.365482	2.345325
O	2.208882	-3.480062	0.373909
O	2.244269	-0.982333	0.590961
C	4.459479	-2.150699	-0.066995

F	4.143106	-2.074410	-1.370882
F	5.129472	-1.045548	0.277872
F	5.246821	-3.215328	0.121120
N	0.183400	-2.135441	-1.261079
H	0.851091	-2.849365	-0.971692
C	-0.917969	-2.611444	-1.795440
N	-1.790846	-1.788742	-2.457278
N	-1.238897	-3.948317	-1.755634
C	-3.229809	-1.861840	-2.202968
H	-3.468279	-2.773034	-1.654040
H	-3.522982	-1.003607	-1.590245
H	-3.789745	-1.854040	-3.146482
C	-1.333682	-0.499132	-2.969231
H	-1.448926	0.296980	-2.229248
H	-0.281132	-0.576172	-3.250116
H	-1.924261	-0.258835	-3.861556
C	-0.507386	-4.851976	-0.874652
H	-0.216714	-4.341408	0.041920
H	-1.159986	-5.692527	-0.615831
H	0.397405	-5.249185	-1.357542
C	-1.925305	-4.604062	-2.864619
H	-1.269186	-5.365387	-3.308493
H	-2.843820	-5.098542	-2.524226
H	-2.178816	-3.874600	-3.633634
P	1.036436	1.978593	-0.230280
C	2.800663	1.807850	-0.857751
C	0.331203	3.363686	-1.246647
C	1.190469	2.557545	1.517492
C	3.897160	2.349325	-0.179496
C	3.001467	1.060722	-2.024473
C	1.183050	4.308447	-1.833988
C	-1.059106	3.482415	-1.386531
C	0.639205	3.774650	1.930918
C	1.804145	1.704354	2.447927
C	5.187284	2.149289	-0.676490
H	3.750905	2.915583	0.734735
C	4.289728	0.863050	-2.516910
H	2.151201	0.609279	-2.531665
C	0.646180	5.371245	-2.561785
H	2.259348	4.215532	-1.726880
C	-1.584832	4.550769	-2.115550
H	-1.711375	2.740228	-0.937637

C	0.699337	4.138868	3.277927
H	0.156023	4.427843	1.210920
C	1.861833	2.078483	3.790052
H	2.216999	0.752629	2.125472
C	5.384934	1.407467	-1.841963
H	6.037757	2.567529	-0.144997
H	4.439460	0.267786	-3.412896
C	-0.737873	5.493376	-2.703441
H	1.309913	6.100525	-3.018173
H	-2.661962	4.642234	-2.226479
C	1.307806	3.292790	4.206914
H	0.266565	5.082226	3.599191
H	2.333397	1.415086	4.509515
H	6.391163	1.242068	-2.216622
H	-1.154548	6.320589	-3.272021
H	1.348583	3.576870	5.254888
C	-2.491917	1.622093	2.078394
H	-2.792332	1.477816	3.122102
H	-1.429561	1.860096	2.043388
H	-3.045623	2.469122	1.662688
C	-2.088335	-0.898360	1.747563
C	-2.629024	-2.161338	1.470769
C	-0.853144	-0.838938	2.409359
C	-1.964507	-3.327598	1.852375
H	-3.580907	-2.232160	0.951485
C	-0.173506	-2.004266	2.775468
H	-0.398797	0.119718	2.627395
C	-0.728404	-3.254443	2.498940
H	-2.409014	-4.295182	1.632689
H	0.801270	-1.935233	3.249235
H	-0.193902	-4.159004	2.774590

TS3'

C	5.808988	0.087119	0.551330
O	6.419695	-0.007504	-0.659515
O	6.343700	-0.251926	1.590394
C	7.749958	-0.544364	-0.623659
H	8.097081	-0.542485	-1.657793
H	8.399744	0.073702	0.002784
H	7.742651	-1.562316	-0.221797
C	4.440444	0.621455	0.405843
H	4.096903	0.896731	-0.584875

C	3.639528	0.738227	1.468767
H	4.036522	0.474060	2.447237
C	2.242191	1.320087	1.409898
C	1.237689	0.524606	2.261296
C	1.526577	-0.737947	2.788233
C	-0.060282	1.030680	2.427295
C	0.549461	-1.466572	3.475271
H	2.505810	-1.181283	2.639065
C	-1.041602	0.304274	3.101222
H	-0.315286	1.986596	1.985669
C	-0.737899	-0.953370	3.630465
H	0.795790	-2.449340	3.869055
H	-2.046659	0.703313	3.186143
H	-1.510110	-1.529393	4.130713
C	2.369737	2.779411	1.943777
H	2.742496	2.779883	2.973607
H	3.069354	3.335309	1.313273
H	1.398578	3.280113	1.918247
Cu	-0.779042	0.062313	-0.310804
O	1.777264	1.471252	0.092085
O	1.075079	-0.120802	-0.396679
S	-3.647863	-0.374633	0.851452
O	-3.734968	-1.263660	2.011264
O	-3.710834	1.082173	1.091753
O	-2.540229	-0.766433	-0.127490
C	-5.117664	-0.763901	-0.220809
F	-5.082165	-0.013802	-1.330814
F	-5.103469	-2.056283	-0.571565
F	-6.246348	-0.503630	0.444663
N	-1.474402	1.894595	-0.631611
H	-2.373762	1.983895	-0.160527
C	-0.885152	3.037578	-0.892905
N	0.133754	3.116248	-1.798774
N	-1.278565	4.219429	-0.295186
C	1.306874	3.955506	-1.561359
H	1.149595	4.582470	-0.683915
H	2.170913	3.311759	-1.366956
H	1.508995	4.594251	-2.430186
C	0.363029	2.013052	-2.726769
H	1.008322	1.247346	-2.284980
H	-0.593352	1.559809	-2.995010
H	0.833294	2.417056	-3.630559
C	-2.159956	4.178640	0.867362
H	-1.921067	3.325486	1.502631
H	-2.017753	5.097529	1.444999
H	-3.220384	4.106374	0.583761
C	-1.359550	5.462636	-1.060846
H	-2.411311	5.752570	-1.195067
H	-0.840681	6.277065	-0.541086
H	-0.912858	5.328442	-2.045618
P	0.552480	-1.948699	-0.937299
O	1.993546	-2.687480	-0.584685
O	0.547552	-2.026210	-2.612665
O	-0.470425	-3.224956	-0.547481
C	3.217540	-2.191158	-1.185082
C	-0.702460	-1.745924	-3.285685
C	-1.006201	-3.324334	0.798871
H	3.231600	-2.503978	-2.235250
H	3.228465	-1.101200	-1.137093
C	4.372328	-2.790349	-0.407138
H	-1.320639	-2.649930	-3.260494
H	-1.253187	-0.951810	-2.760510
C	-0.369827	-1.318869	-4.702488
H	-0.276389	-3.867739	1.410635
H	-1.146886	-2.327852	1.226140
C	-2.333439	-4.049894	0.714834
H	5.318404	-2.472876	-0.858122
H	4.353970	-2.447404	0.631571
H	4.321724	-3.883568	-0.419572
H	0.203588	-2.099237	-5.212573
H	-1.291552	-1.136259	-5.266357
H	0.226458	-0.400480	-4.694408
H	-2.205815	-5.043698	0.272883
H	-2.756937	-4.156108	1.719129
H	-3.037374	-3.472904	0.111015

TS4

C	0.089429	1.693104	-3.158469
O	-1.186788	2.125551	-3.296097
O	0.546233	0.729227	-3.737086
C	-2.017718	1.310138	-4.156021
H	-2.971588	1.834313	-4.218544
H	-1.563029	1.217513	-5.144508
H	-2.142938	0.319131	-3.711787

C	0.778448	2.489491	-2.111082
H	0.364400	3.465192	-1.873483
C	1.730579	1.899056	-1.378612
H	2.060479	0.909452	-1.681441
C	2.207980	2.337389	-0.002050
C	3.568637	1.729698	0.335659
C	4.552628	1.577335	-0.648134
C	3.855590	1.338955	1.650527
C	5.794552	1.023222	-0.330213
H	4.351857	1.889265	-1.670487
C	5.096895	0.782339	1.968944
H	3.097635	1.465588	2.416929
C	6.068195	0.617374	0.977727
H	6.546725	0.908883	-1.105485
H	5.307908	0.487020	2.993412
H	7.034103	0.186525	1.224932
C	2.248647	3.858854	0.195069
H	2.989167	4.314785	-0.468717
H	1.271038	4.311382	-0.003175
H	2.526655	4.077901	1.229661
Cu	0.207760	0.151839	0.425117
O	1.219889	1.803031	0.910280
S	-2.050373	-1.787660	-0.264853
O	-2.187900	-0.955529	-1.475969
O	-1.621827	-3.175331	-0.418744
O	-1.295937	-1.035935	0.843059
C	-3.754993	-1.882487	0.494280
F	-3.676255	-2.277001	1.766552
F	-4.342729	-0.665271	0.464184
F	-4.501191	-2.731717	-0.199932
N	1.762922	-0.966914	0.258358
H	2.509238	-0.561200	0.817445
C	1.987356	-2.248902	-0.036394
N	1.410074	-2.833861	-1.106880
N	2.841156	-2.991042	0.732908
C	1.124609	-4.268729	-1.168685
H	1.215269	-4.710702	-0.176428
H	0.094707	-4.395919	-1.506445
H	1.801448	-4.780826	-1.862901
C	0.978666	-2.065022	-2.275187
H	-0.109702	-2.007295	-2.335669
H	1.384077	-1.057753	-2.239821
H	1.353182	-2.560583	-3.177869
C	3.095944	-2.618546	2.122026
H	2.200578	-2.167741	2.555518
H	3.339590	-3.523331	2.687194
H	3.935367	-1.914539	2.211712
C	3.820094	-3.906503	0.142766
H	4.826459	-3.605125	0.457178
H	3.647121	-4.940133	0.462828
H	3.774830	-3.856564	-0.945121
N	-1.132705	1.941182	0.357407
H	-1.282285	2.246821	-0.600407
C	-2.257933	1.901078	1.118658
N	-2.148050	1.723006	2.456801
N	-3.465017	2.029617	0.545288
C	-3.017439	0.778086	3.177521
H	-3.773430	0.363799	2.517532
H	-2.401431	-0.055509	3.532308
H	-3.494128	1.267101	4.032411
C	-0.886960	2.004978	3.143529
H	-0.189263	1.159898	3.076327
H	-0.406436	2.891210	2.729801
H	-1.112309	2.189791	4.196887
C	-3.639616	1.888300	-0.906727
H	-2.973330	1.120747	-1.299086
H	-4.663975	1.560026	-1.091450
H	-3.473042	2.843515	-1.419209
C	-4.656409	2.460705	1.282236
H	-5.142071	3.256759	0.709587
H	-5.362910	1.633372	1.403798
H	-4.377908	2.855424	2.258969
H	-0.000668	2.185438	0.714696

TS5

C	4.203833	1.695565	-0.430948
O	5.530481	1.959546	-0.318269
O	3.347118	2.503974	-0.103942
C	5.853143	3.252442	0.208429
H	6.942922	3.300269	0.227511
H	5.444664	4.043619	-0.427604
H	5.446638	3.372858	1.217736
C	3.977382	0.336879	-0.958839
H	4.856945	-0.274919	-1.131083

C	2.725508	-0.090283	-1.173049
H	1.915022	0.601364	-0.984475
C	2.240095	-1.440794	-1.657110
C	1.719338	-2.270869	-0.459430
C	2.223641	-2.145830	0.838967
C	0.712537	-3.212692	-0.701670
C	1.721128	-2.936612	1.875828
H	2.994809	-1.411884	1.051188
C	0.207915	-4.002096	0.330682
H	0.329269	-3.297702	-1.713265
C	0.709176	-3.864543	1.628278
H	2.112855	-2.812037	2.882129
H	-0.583525	-4.719392	0.125711
H	0.312691	-4.473335	2.437120
C	3.358284	-2.249749	-2.359934
H	2.940603	-3.193544	-2.720140
H	3.746381	-1.683666	-3.212130
H	4.177719	-2.474129	-1.667727
O	-0.115070	-0.313513	-1.660775
O	1.250196	-1.245868	-2.618485
H	-0.239183	0.163357	-2.500662
P	-1.541385	0.545393	-0.419917
C	-0.536631	0.511315	1.132915
C	-2.500588	2.145581	-0.407133
C	-2.825724	-0.782457	-0.280233
C	-0.858189	-0.331210	2.202360
C	-2.129183	3.232204	0.395989
C	-4.006419	-0.590007	0.452098
C	0.610492	1.314886	1.199168
C	-3.572868	2.283144	-1.303344
C	-2.583481	-2.007969	-0.911358
C	-0.041607	-0.358266	3.334059
C	-2.831857	4.435661	0.310320
C	-4.937229	-1.624311	0.552942
C	1.424169	1.281882	2.330598
C	-4.274491	3.484783	-1.379206
C	-3.518171	-3.039188	-0.803515
C	1.096666	0.447280	3.401517
C	-3.904386	4.565835	-0.573842
C	-4.693122	-2.850004	-0.073436
H	-1.727709	-0.977889	2.147976
H	-1.303613	3.139128	1.093932
H	-4.198494	0.362666	0.936910
H	0.902263	1.951068	0.370150
H	-3.866989	1.445431	-1.930521
H	-1.667195	-2.139650	-1.474109
H	-0.290191	-1.022588	4.157004
H	-2.541612	5.270451	0.942570
H	-5.850958	-1.473534	1.121427
H	2.320059	1.894653	2.346209
H	-5.109419	3.577897	-2.068442
H	-3.326839	-3.989516	-1.294341
H	1.734266	0.413811	4.281066
H	-4.448865	5.503879	-0.636956
H	-5.419717	-3.654143	0.006699

TSA1

C	-2.683777	-0.298507	0.156583
O	-3.143522	-1.385577	-0.508378
O	-3.364977	0.357692	0.928093
C	-4.514019	-1.721455	-0.247001
H	-4.719106	-2.608284	-0.847765
H	-4.663614	-1.932532	0.816235
H	-5.174031	-0.898321	-0.536830
C	-1.275897	-0.041141	-0.160606
H	-0.769615	-0.691891	-0.864095
C	-0.646181	1.073201	0.372561
H	-1.171382	1.609461	1.158561
C	0.861539	1.345290	0.183680
C	1.723559	0.100836	0.086167
C	1.872023	-0.721869	1.211628
C	2.364107	-0.249135	-1.106406
C	2.654764	-1.873293	1.146680
H	1.363604	-0.469518	2.138920
C	3.154388	-1.400447	-1.167244
H	2.238061	0.375797	-1.983450
C	3.302895	-2.214038	-0.044005
H	2.758271	-2.504600	2.025005
H	3.649809	-1.661044	-2.098627
H	3.915029	-3.110285	-0.094549
C	1.396854	2.359151	1.193357
H	2.415304	2.650368	0.921074
H	0.756837	3.247071	1.189521
H	1.414587	1.935988	2.203213

O	-0.644547	2.283033	-1.024417
O	0.743636	1.983131	-1.117747

TSA2

C	2.483040	0.051078	-0.155161
O	3.019761	1.185441	-0.670157
O	2.930566	-0.514068	0.832463
C	4.168741	1.683176	0.029204
H	4.475890	2.580048	-0.510219
H	3.915335	1.925599	1.065920
H	4.972447	0.940574	0.030922
C	1.309700	-0.375034	-0.914072
H	0.922502	0.258594	-1.702883
C	0.630307	-1.509096	-0.514692
H	1.147720	-2.222791	0.128725
C	-0.996426	-1.365525	0.520565
C	-1.602391	-0.042154	0.303786
C	-1.403183	1.000781	1.228149
C	-2.336438	0.219058	-0.868154
C	-1.933982	2.266164	0.990471
H	-0.825883	0.825577	2.130626
C	-2.867516	1.485684	-1.098375
H	-2.486035	-0.581712	-1.582742
C	-2.668892	2.514170	-0.172633
H	-1.773522	3.061161	1.713620
H	-3.439585	1.671558	-2.003376
H	-3.082332	3.502051	-0.356199
C	-0.781523	-1.882372	1.917801
H	-1.703684	-1.830511	2.510741
H	-0.444103	-2.920548	1.877425
H	-0.013811	-1.290661	2.428670
O	-0.293680	-2.078479	-1.419290
O	-1.365842	-2.367210	-0.409587

TSB1

C	-1.846868	0.356664	2.799925
O	-1.480898	-0.416664	3.877791
O	-2.013583	1.573586	2.917778
C	-1.287554	0.301784	5.100131
H	-1.001477	-0.447363	5.841103
H	-0.497516	1.052009	4.991910
H	-2.207526	0.808299	5.407152
C	-1.962595	-0.415412	1.597395
H	-1.747069	-1.471982	1.639759
C	-2.488506	0.176914	0.443380
H	-2.927304	1.160507	0.587371
C	-3.024057	-0.635382	-0.756630
C	-3.328590	-2.111908	-0.508171
H	-3.551939	-2.597705	-1.463896
H	-2.476862	-2.618872	-0.048806
H	-4.202145	-2.221257	0.141311
C	-4.221183	0.060991	-1.396621
C	-5.384645	0.269855	-0.643654
C	-4.182706	0.485020	-2.727154
C	-6.496040	0.889377	-1.214920
H	-5.415991	-0.044450	0.397799
C	-5.296923	1.103380	-3.299956
H	-3.273546	0.336661	-3.299903
C	-6.455737	1.305970	-2.548389
H	-7.391274	1.048935	-0.619438
H	-5.255870	1.430709	-4.335710
H	-7.320747	1.788671	-2.995518
Cu	0.487410	0.107879	0.304242
S	2.782346	0.029563	-2.124237
O	2.108452	-1.198121	-2.598509
O	2.015941	0.774173	-1.056932
O	3.389791	0.912183	-3.121828
C	4.226274	-0.608191	-1.130487
F	4.999050	0.413655	-0.713834
F	4.978463	-1.446240	-1.847943
F	3.797749	-1.265034	-0.026760
N	0.400097	1.797095	1.224593
H	-0.546778	2.022724	1.531102
C	1.079300	2.866394	0.828412
N	2.439117	2.864307	0.858267
N	0.442761	3.997704	0.406940
C	3.236343	3.449779	-0.222102
H	2.581320	3.885737	-0.975687
H	3.815615	2.659544	-0.706127
H	3.916072	4.219271	0.164361
C	3.133004	1.866658	1.666321
H	3.286788	0.938085	1.110164
H	2.549778	1.653931	2.563347
H	4.109341	2.272921	1.951832

C	-0.941016	3.918194	-0.060040
H	-1.114649	2.957588	-0.551551
H	-1.107079	4.718726	-0.787944
H	-1.658313	4.039338	0.763900
C	0.965347	5.336819	0.650537
H	0.220734	5.923705	1.203560
H	1.190498	5.858556	-0.288047
H	1.874668	5.277215	1.249573
N	0.709460	-1.737500	-0.158828
H	0.936123	-1.824817	-1.150497
C	0.415604	-2.852415	0.453386
N	0.484221	-2.938362	1.820591
N	0.025930	-3.981308	-0.234926
C	-0.498669	-3.673340	2.613943
H	-1.225038	-4.153479	1.956410
H	-1.027420	-2.975864	3.275135
H	-0.014719	-4.442267	3.230185
C	1.278725	-1.966053	2.564593
H	0.680243	-1.098409	2.867369
H	2.120616	-1.637157	1.952789
H	1.663639	-2.451587	3.468943
C	-0.325163	-3.847882	-1.649332
H	-0.866525	-2.914632	-1.813857
H	-0.969821	-4.688160	-1.925977
H	0.565068	-3.859032	-2.294653
C	0.532314	-5.301309	0.138902
H	1.284451	-5.636787	-0.588996
H	-0.281827	-6.035109	0.158814
H	1.001493	-5.263524	1.121637
O	-1.248971	0.575614	-0.802540
O	-1.847714	-0.528599	-1.575836

TSB2

C	-1.055193	-3.159972	-0.142771
O	-2.304910	-3.375532	-0.655838
O	-0.103441	-3.852261	-0.488811
C	-2.388509	-4.432438	-1.623198
H	-3.438183	-4.477312	-1.919173
H	-1.756020	-4.216296	-2.489908
H	-2.073337	-5.383510	-1.186037
C	-1.008005	-2.028407	0.794408
H	-1.996808	-1.714598	1.126708
C	-0.102517	-2.321687	1.967197
H	0.722471	-2.978887	1.666037
C	0.605451	-1.145802	2.858117
C	-0.312464	0.076444	2.955431
H	-0.415880	0.600239	2.004181
H	-1.294249	-0.263562	3.294641
H	0.094192	0.779771	3.688422
C	2.033713	-0.860145	2.421785
C	3.033596	-1.802418	2.723916
C	2.399722	0.323378	1.770544
C	4.362988	-1.557378	2.385610
H	2.756711	-2.709581	3.250987
C	3.729130	0.562661	1.413171
H	1.659618	1.068857	1.530635
C	4.715494	-0.372804	1.727776
H	5.126206	-2.288930	2.639069
H	3.966851	1.487892	0.896700
H	5.753552	-0.183210	1.465487
Cu	-0.512827	-0.387757	-0.497549
S	0.952481	2.927882	-0.588443
O	2.368703	3.180539	-0.285105
O	0.552392	1.497412	-0.376236
O	0.395788	3.526004	-1.814053
C	0.026364	3.760589	0.800058
F	-1.315073	3.626612	0.614748
F	0.303016	5.061898	0.866538
F	0.323791	3.189628	1.980042
N	0.854241	-1.315231	-1.502713
H	1.021227	-2.266223	-1.180451
C	1.871884	-0.793282	-2.166917
N	1.692815	0.271277	-2.995828
N	3.132679	-1.317404	-2.069005
C	2.703736	1.322909	-3.138223
H	3.426317	1.256165	-2.323893
H	2.210101	2.294496	-3.068822
H	3.226151	1.259980	-4.101434
C	0.361117	0.597724	-3.498486
H	-0.131653	1.342011	-2.865480
H	-0.241685	-0.311234	-3.537985
H	0.461189	1.008808	-4.509247
C	3.477751	-2.175192	-0.940556
H	2.921825	-1.869991	-0.055446

H	4.542635	-2.061115	-0.722398
H	3.271286	-3.234449	-1.153517
C	4.041621	-1.377633	-3.208492
H	4.372595	-2.413916	-3.352352
H	4.927239	-0.748828	-3.054522
H	3.529963	-1.053707	-4.115462
N	-2.109210	0.703994	-0.166338
H	-1.872046	1.581415	0.284764
C	-3.396104	0.451624	-0.185348
N	-3.913043	-0.432200	-1.092272
N	-4.280963	1.051544	0.680903
C	-4.958181	-1.388696	-0.735334
H	-5.332277	-1.174933	0.266199
H	-4.539337	-2.400631	-0.737376
H	-5.792420	-1.337969	-1.446182
C	-3.177235	-0.727051	-2.316645
H	-2.492539	-1.571795	-2.188116
H	-2.605190	0.152009	-2.617663
H	-3.899061	-0.974156	-3.103761
C	-3.775952	1.685427	1.894933
H	-2.956134	1.094920	2.307954
H	-4.584632	1.721381	2.631412
H	-3.420124	2.710002	1.716766
C	-5.586412	1.528065	0.229076
H	-5.607195	2.626636	0.232409
H	-6.384720	1.164187	0.886939
H	-5.778524	1.183439	-0.786987
O	0.613578	-1.843780	4.062857
O	-0.871989	-2.873774	2.984615

TSB3

C	2.133636	-0.153573	2.458856
O	2.015985	0.688283	3.530357
O	2.350037	-1.356477	2.595068
C	2.130900	0.057697	4.813210
H	2.007736	0.856819	5.545755
H	1.354862	-0.703280	4.940760
H	3.109742	-0.417772	4.925445
C	1.939077	0.534776	1.207249
H	1.723328	1.591405	1.222464
C	2.194374	-0.116602	-0.069830
H	2.475640	-1.164629	0.072984
C	3.061278	0.508458	-1.211548
C	3.294204	2.015242	-1.067446
H	3.716567	2.409432	-1.997288
H	2.344395	2.521811	-0.875339
H	3.986700	2.238605	-0.248211
C	4.349249	-0.257285	-1.450734
C	5.362670	-0.239140	-0.482157
C	4.534318	-1.010090	-2.614777
C	6.544014	-0.953998	-0.678209
H	5.220078	0.324821	0.436810
C	5.721328	-1.718935	-2.815289
H	3.739936	-1.038827	-3.353261
C	6.729354	-1.692985	-1.849986
H	7.318675	-0.936298	0.084153
H	5.855567	-2.295676	-3.726966
H	7.651016	-2.247587	-2.005502
Cu	-0.666135	0.008937	0.475176
S	-3.180834	-0.141812	-1.797722
O	-2.663997	1.147043	-2.311997
O	-2.263476	-0.799598	-0.799571
O	-3.778349	-1.074588	-2.754917
C	-4.591142	0.371753	-0.691607
F	-5.251171	-0.708422	-0.226330
F	-5.460510	1.151056	-1.340071
F	-4.125470	1.052124	0.378987
N	-0.372400	-1.704878	1.308283
H	0.614579	-1.904906	1.461698
C	-1.035107	-2.789445	0.913575
N	-2.371770	-2.893371	1.121506
N	-0.391755	-3.830786	0.307849
C	-3.258397	-3.508460	0.129713
H	-2.687331	-3.809147	-0.747644
H	-3.991333	-2.768043	-0.196553
H	-3.778436	-4.377479	0.551129
C	-3.028190	-1.974803	2.046735
H	-3.323120	-1.049464	1.542178
H	-2.350571	-1.734995	2.866966
H	-3.924761	-2.463843	2.442253
C	0.863402	-3.587886	-0.401267
H	0.855885	-2.582244	-0.827944
H	0.946737	-4.311949	-1.218072
H	1.737752	-3.702006	0.255115

C	-0.758258	-5.224873	0.523183
H	0.116421	-5.779092	0.887331
H	-1.108923	-5.699685	-0.401777
H	-1.545930	-5.289231	1.274901
N	-1.109729	1.833275	0.036237
H	-1.556983	1.816605	-0.884085
C	-0.458066	2.947346	0.270268
N	-0.111367	3.283999	1.552644
N	-0.100060	3.808503	-0.738799
C	1.099101	4.030154	1.883292
H	1.680951	4.220242	0.980343
H	1.712124	3.439792	2.575913
H	0.861504	4.988095	2.363897
C	-0.748978	2.600310	2.673468
H	-0.169179	1.724583	2.989583
H	-1.749289	2.279552	2.380745
H	-0.819212	3.297667	3.516120
C	-0.077478	3.303571	-2.116806
H	0.333013	2.291674	-2.140797
H	0.573171	3.958868	-2.704154
H	-1.077029	3.293927	-2.570406
C	-0.275549	5.253379	-0.609771
H	-1.085070	5.591627	-1.270884
H	0.642250	5.785909	-0.886703
H	-0.541937	5.513328	0.415127
O	0.971401	-0.002773	-0.830931
O	2.083552	0.253551	-2.213091

P1

C	-2.870201	-0.259386	-0.304634
O	-3.886517	-0.435091	0.576342
O	-2.942058	-0.580651	-1.476326
C	-5.068044	-1.034535	0.026906
H	-5.777214	-1.100282	0.853220
H	-5.474267	-0.418286	-0.780939
H	-4.847135	-2.029773	-0.370804
C	-1.704595	0.363495	0.360533
H	-1.784802	0.602674	1.414348
C	-0.594301	0.615523	-0.339638
H	-0.568347	0.351049	-1.394908
C	0.664182	1.241928	0.207866
C	1.803432	0.217329	0.062715
C	1.899936	-0.810292	1.013310
C	2.725760	0.248868	-0.990013
C	2.902889	-1.773641	0.921921
H	1.179808	-0.839685	1.824900
C	3.729905	-0.718596	-1.083861
H	2.674391	1.027173	-1.744492
C	3.823686	-1.730801	-0.128566
H	2.964048	-2.560405	1.669276
H	4.440113	-0.676383	-1.905435
H	4.605852	-2.481548	-0.201915
C	0.933717	2.563910	-0.536359
H	0.951258	2.435620	-1.623021
H	0.137106	3.269069	-0.285429
H	1.895891	2.986844	-0.224098
O	0.433610	1.527860	1.591767
H	1.284823	1.794180	1.973954

P2

C	-1.698143	-0.204411	0.000040
O	-2.215571	-1.311353	-0.000143
C	-2.558237	1.049683	0.000173
H	-3.608631	0.753772	0.000255
H	-2.352811	1.665978	0.883719
H	-2.353057	1.665983	-0.883422
C	-0.205888	-0.054702	-0.000061
C	0.431352	1.195787	-0.000138
C	0.576845	-1.220740	-0.000021
C	1.824225	1.277611	-0.000070
H	-0.153226	2.110551	-0.000210
C	1.966019	-1.139091	0.000097
H	0.068235	-2.179349	0.000038
C	2.592875	0.111566	0.000063
H	2.308953	2.250048	-0.000113
H	2.563087	-2.046963	0.000246
H	3.677740	0.176585	0.000139
